# Interrogating the topological robustness of gene regulatory circuits by randomization

**DOI:** 10.1371/journal.pcbi.1005456

**Published:** 2017-03-31

**Authors:** Bin Huang, Mingyang Lu, Dongya Jia, Eshel Ben-Jacob, Herbert Levine, Jose N. Onuchic

**Affiliations:** 1 Center for Theoretical Biological Physics, Rice University, Houston, TX, United States of America; 2 Department of Chemistry, Rice University, Houston, TX, United States of America; 3 The Jackson Laboratory, Bar Harbor, ME, United States of America; 4 Program in Systems, Synthetic and Physical Biology, Rice University, Houston, TX, United States of America; 5 School of Physics and Astronomy, and The Sagol School of Neuroscience, Tel-Aviv University, Tel-Aviv, Israel; 6 Department of Bioengineering, Rice University, Houston, TX, United States of America; 7 Department of Biosciences, Rice University, Houston, TX, United States of America; 8 Department of Physics and Astronomy, Rice University, Houston, TX, United States of America; Peking University, CHINA

## Abstract

One of the most important roles of cells is performing their cellular tasks properly for survival. Cells usually achieve robust functionality, for example, cell-fate decision-making and signal transduction, through multiple layers of regulation involving many genes. Despite the combinatorial complexity of gene regulation, its quantitative behavior has been typically studied on the basis of experimentally verified core gene regulatory circuitry, composed of a small set of important elements. It is still unclear how such a core circuit operates in the presence of many other regulatory molecules and in a crowded and noisy cellular environment. Here we report a new computational method, named random circuit perturbation (RACIPE), for interrogating the robust dynamical behavior of a gene regulatory circuit even without accurate measurements of circuit kinetic parameters. RACIPE generates an ensemble of random kinetic models corresponding to a fixed circuit topology, and utilizes statistical tools to identify generic properties of the circuit. By applying RACIPE to simple toggle-switch-like motifs, we observed that the stable states of all models converge to experimentally observed gene state clusters even when the parameters are strongly perturbed. RACIPE was further applied to a proposed 22-gene network of the Epithelial-to-Mesenchymal Transition (EMT), from which we identified four experimentally observed gene states, including the states that are associated with two different types of hybrid Epithelial/Mesenchymal phenotypes. Our results suggest that dynamics of a gene circuit is mainly determined by its topology, not by detailed circuit parameters. Our work provides a theoretical foundation for circuit-based systems biology modeling. We anticipate RACIPE to be a powerful tool to predict and decode circuit design principles in an unbiased manner, and to quantitatively evaluate the robustness and heterogeneity of gene expression.

## Author summary

Cells are able to robustly carry out their essential biological functions, possibly because of multiple layers of tight regulation via complex, yet well-designed, gene regulatory networks involving a substantial number of genes. State-of-the-art genomics technology has enabled the mapping of these large gene networks, yet it remains a tremendous challenge to elucidate their design principles and the regulatory mechanisms underlying their biological functions such as signal processing and decision-making. One of the key barriers is the absence of accurate kinetics for the regulatory interactions, especially from *in vivo* experiments. To this end, we have developed a new computational modeling method, Random Circuit Perturbation (RACIPE), to explore the dynamic behaviors of gene regulatory circuits without the requirement of detailed kinetic parameters. RACIPE takes a network topology as the input, and generates an unbiased ensemble of models with varying kinetic parameters. Each model is subjected to simulation, followed by statistical analysis for the ensemble. We tested RACIPE on several gene circuits, and found that the predicted gene expression patterns from all of the models converge to experimentally observed gene state clusters. We expect RACIPE to be a powerful method to identify the role of network topology in determining network operating principles.

## Introduction

State-of-the-art molecular profiling techniques[1–4] have enabled the construction or inference of large gene regulatory networks underlying certain cellular functions, such as cell differentiation[5,6] and circadian rhythm[7,8]. However, it remains a challenge to understand the operating principles of these regulatory networks and how they can robustly perform their tasks, a prerequisite for cell survival. Mathematical and computational systems biology approaches are often applied to quantitatively model the dynamic behaviors of a network[9–20]. Yet, quantitative simulations of network dynamics are usually limited due to several reasons. First, a proposed network might contain inaccurate or missing regulatory genes or links, and modeling an incomplete network might produce inaccurate predictions. Second, kinetic parameters for each gene and regulatory interaction, which are usually required for quantitative analyses, are difficult to obtain altogether directly from *in vivo* experiments[21]. To deal with this problem, network parameters are either inferred from existing data [22,23] or educated guesses, an approach which could be time-consuming and error-prone. This approach is hard to extend to very large gene networks due to their complexity.

Alternatively, a bottom-up strategy has been widely used to study the regulatory mechanisms of cellular functions. First, one performs a comprehensive analysis and integration of experimental evidence for the essential regulatory interactions in order to construct a core regulatory circuit, typically composed of only a small set of essential genes. The core gene circuit is then modeled either by deterministic or stochastic approaches with a particular set of parameters inferred from the literature. Due to the reduced size of the systems and the inclusion of data derived directly from the literature, the bottom-up approach suffers less from the above-mentioned issues. Examples of the bottom-up approach include the modeling of biological processes such as Epithelial-to-Mesenchymal Transition (EMT)[24–26], cell cycles[27,28], and circuit designs in synthetic biology, such as genetic toggle switch[29] and repressilator[30].

Due to the success of these and other circuit-based modeling studies, we hypothesize that a core circuit module should emerge from a complex network and dictate the decision-making process. It is reasonable to assume that a large gene network could be decomposed into a core gene circuit and a peripheral part with the residual genes. The core would then be the driving force for the network dynamics and should be robust against cell-to-cell variability and extrinsic fluctuations in stimuli arising from cell signaling. While the peripheral genes would either act to regulate the signaling status for the core circuit and probably also enhance the robustness of the core dynamics by introducing redundancy (in-components–genes that regulate the core unit) or simply have no regulatory effects on the core (e.g. out-components–genes that are regulated by the core unit). This scale-separation picture is consistent with ideas such as the existence of master regulators and network modularity[31,32].

On the basis of this conceptual framework, we developed a new computational method, named *ra*ndom *ci*rcuit *pe*rturbation (RACIPE), for modeling possible dynamic behaviors that are defined by the topology of a core gene regulatory circuit. In RACIPE, we focus the modeling analysis on the core circuit and regard the effects of the peripheral genes and external signaling as random perturbations to the kinetic parameters. In contrast to traditional modeling methods[33], RACIPE generates an ensemble of mathematical models, each of which has a different set of kinetic parameters representing variations of signaling states, epigenetic states, and genetic backgrounds (including cells with genetic mutations leading to disease). Here we randomize the model parameters by one or two orders of magnitude and utilize a specially designed sampling scheme (details in Methods) to capture the key role of the circuit topology. This random field approach allows the inclusion of the contributions from the peripheral genes to the network dynamics and the evaluation of their roles in modulating the functions of the core circuit. From the *in silico* generated data, we apply statistical analysis to identify the most probable features within all of the models, a process which can uncover the most robust functions of the core circuit. It is worth-noting that RACIPE is unique in the way it utilizes perturbation and the integration of statistical tools, compared to the traditional parameter sensitivity analysis[34–38] and the previous studies on random circuit topology[39,40].

In the following, we will first describe in detail the RACIPE method, and then present the results of applying RACIPE to several simple standalone circuit motifs and also coupled toggle switch motifs. In addition, we will show the application of RACIPE to a 22-component network for the decision-making core of the Epithelial-to-Mesenchymal Transition (EMT). We will see that RACIPE is capable of identifying accessible gene states via statistical analysis of the *in silico* generated data, from which we can further decode the design principles and evaluate the robustness of the core gene circuit. We therefore expect RACIPE to be a powerful tool to analyze the dynamic behavior of a gene network and to evaluate the robustness and accuracy of proposed network models.

## Methods

**Fig 1:**
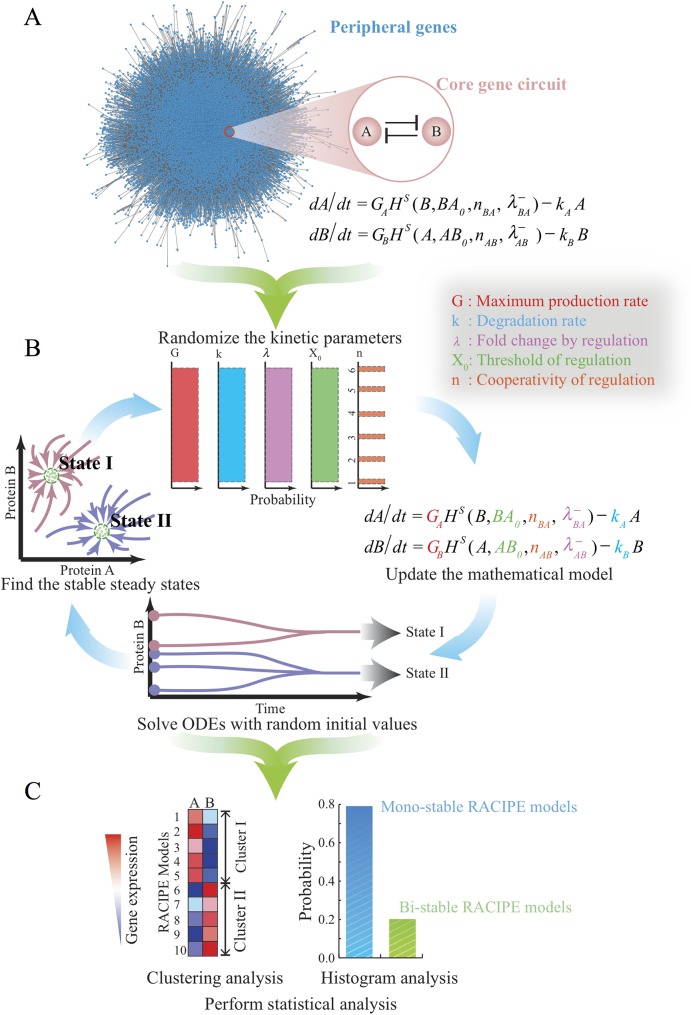
Schematics of the random circuit perturbation (RACIPE) method. **(A)** The gene regulatory network for a specific cellular function is decomposed into two parts–a core gene circuit modeled by chemical rate equations and the other peripheral genes whose contribution to the network is regarded as random perturbations to the kinetic parameters of the core circuit; **(B)** RACIPE generates an ensemble of models, each of which is simulated by the same rate equations but with randomly sampled kinetic parameters. For each model, multiple runs of simulations are performed, starting from different initial conditions, to identify all possible stable steady states; **(C)** The *in silico* gene expression data derived from all of the models are subject to statistical analysis.

We developed a new computational method, namely *ra*ndom *ci*rcuit *pe*rturbation (RACIPE), for modeling a gene network. The procedure of RACIPE is illustrated in Fig 1. The input of RACIPE is the topology of the core circuit under study, which can be constructed according to either the literature, interaction databases (e.g. Ingenuity pathway analysis (IPA®, QIAGEN Redwood City, www.qiagen.com/ingenuity), KEGG[41], GO[42]), or computational methods[43]. From the circuit topology, we establish a set of mathematical equations for the time evolution of the levels of all the genes. We then generate an ensemble of models where the parameters of the rate equations are sampled by a carefully designed randomization procedure (see below for details) so that these kinetic models can capture the behavior of the circuits under different conditions. Each model is subject to standard analysis to discover possible dynamics of the circuit (Fig 1B). The dynamics could converge to a stable steady state, a stable oscillation, or chaotic behaviors. To find all possible behaviors of a gene network, we typically choose many different sets of initial conditions (randomly sampled on a logarithmic scale) and numerically solve the rate equations for each case. The procedure is repeated for many times to collect sufficient data for statistical analysis. In particular, for a multi-stable system, this ODE-based method is useful for identifying all the distinct stable states for a multi-stable system. Thus, the RACIPE method can generate a large amount of simulated gene expression data, which can be further analyzed by biostatistical tools (Fig 1C). Potentially, RACIPE can be further extended to study oscillatory (S1 Fig) or adaptive dynamics, and is also compatible with other types of modeling methods such as stochastic analysis, but these are out of scope of this study. In the following, we will illustrate RACIPE in the context of a multi-stable gene circuit by deterministic analysis.

**Fig 2:**
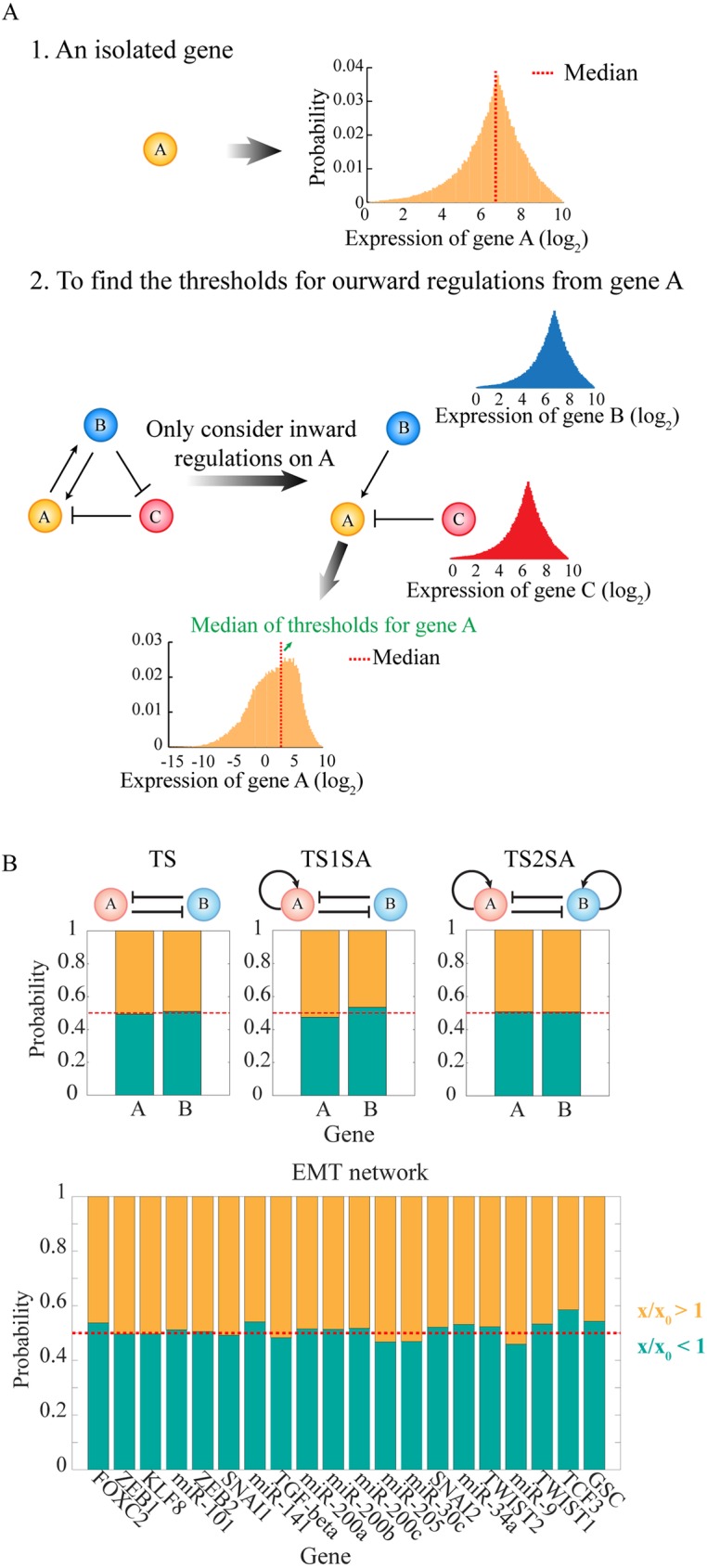
Randomization scheme to estimate the ranges of the threshold parameters. **(A)** Schematic of the procedure to estimate the ranges of the threshold parameters, so that the level of a regulator has 50% chance to be above or below the threshold level of each regulatory link (“half-functional rule”). First, for a gene A without any regulator, the RACIPE models are generated by randomizing the maximum production rate and the degradation rate according to S1 Table. The distribution of A level is obtained from the stable steady state solutions of all the RACIPE models (top left panel, yellow histogram). Second, for a gene A in a gene circuit, the distribution of A level is estimated only on the basis of the inward regulatory links (i.e. the B to A activation and the C to A inhibition in the bottom left panel). The distributions of the levels of the inward regulators B and C are assumed to follow the same distributions as a gene without any regulator (bottom left panel, blue and red distribution); the threshold levels for these inward links are chosen randomly from (0.02M to 1.98M), where M is the median of their gene expression distributions. Finally, the distribution of A level is obtained by randomizing all the relevant parameters. That includes the levels of B and C, the strength of the inward regulatory links (i.e., the threshold level, the Hill coefficient and the fold change), the maximum production rate and the degradation rate of A, and the threshold for any regulatory link starting from A is chosen randomly from (0.02M to 1.98M), where M is the median level of the new distribution of A level (orange in the bottom panel). The same procedure is followed for all other genes. **(B)** Tests on several simple toggle-switch-like circuit motifs and the Epithelial-to-Mesenchymal Transition (EMT) circuit show that the “half-functional rule” is approximately satisfied with this randomization scheme. For each RACIPE model, we computed the ratio (x/x_0_) of the level of each gene X at each stable steady state (x) and the threshold (x_0_) for each outward regulations from gene X. The yellow region shows the probability of x/x_0_ > 1 for all the RACIPE models, and the green region shows the probability of x/x_0_ < 1.

As an example, we start with the deterministic rate equations for a toggle switch circuit (Fig 2) with mutually inhibitory genes A and B. The kinetic model takes the form:

$$\begin{array}{l} \dot{A}=g_{A}H^{S}(B,BA_{0},n_{BA},\lambda_{BA}^{-})-k_{A}A\\ \dot{B}=g_{B}H^{S}(A,AB_{0},n_{AB},\lambda_{AB}^{-})-k_{B}B \end{array},$$ (1)

where *A* and *B* represent the expression levels of gene A and B respectively. *g*_*A*_ and *g*_*B*_ are the basal production rates (the production rates of the genes without any regulator bound to the promoter). *k*_*A*_ and *k*_*B*_ are the innate degradation rates. Regulation of gene B expression by A is formulated as a non-linear shifted Hill function $H^{S}(A,AB_{0},n_{AB},\lambda_{AB}^{-})$, defined as $\lambda_{AB}^{-}+(1-\lambda_{AB}^{-})H^{-}(A,AB_{0},n_{AB})$, where $H^{-}=1/(1+{\left(A/AB_{0}\right)}^{n_{AB}})$ is the inhibitory Hill function, *AB*_0_ is the threshold level for A, *n*_*AB*_ is the Hill coefficient of the regulation, $\lambda_{AB}^{-}$ is the maximum fold change of the B level caused by the inhibitor A ($0\leq \lambda_{AB}^{-}<1$). In the case of an activator, the fold change is represented by $\lambda_{AB}^{+}$ ($\lambda_{AB}^{+}>1$). The inhibitory regulation of gene A by gene B can be modeled in an analogous way.

In RACIPE, randomization is performed on all five types of circuit parameters: two of them are associated with each gene, including the basal production rate (*g*) and the degradation rate (*k*); and three of them are associated with each regulatory link, including the maximum fold change of the gene expression level (*λ*), the threshold level of the regulation (*X*_0_) and the Hill coefficient (n).

Our parametric randomization procedure is designed to ensure that the models can represent all biologically relevant possibilities. In detail, the Hill coefficient *n* is an integer selected from 1 to 6, and the degradation rate *k* ranges from 0.1 to 1 (See S1 Table for the explanation of the units). Here each parameter is assigned by randomly picking values from either a uniform distribution or some other distributions, for example the Gaussian distribution. In this work, we mainly used uniform distribution for sampling parameters unless other distributions are explicitly mentioned. The fold change *λ*^+^ ranges from 1 to 100 if the regulatory link is excitatory, while *λ*^−^ was varied from 0.01 to 1 if the regulatory link is inhibitory. Note that for the latter case, a probability distribution (e.g. a uniform distribution) is sampled for the inverse of *λ*^−^, i.e. 1/*λ*^−^, instead of *λ*^−^ itself. By doing so, we make sure that the mean fold change is about 0.02, instead of ~ 0.5. The choice of such a wide range of *λ* values ensures the consideration of both strong and weak interactions.

In addition, two assumptions are made in RACIPE to ensure that it generates a representative ensemble of models for a specific circuit topology. First, the maximum production rate of each gene should lie roughly within the same range (from 1 to 100 in this study, see S1 Table), as the maximum rate is determined by how fastest the transcriptional machinery can work. For a gene regulated by only one activator, the maximum production rate (*G*) is achieved when the activator is abundant, and thus the basal production rate of the gene *g* = *G*/*λ*^+^. For a gene regulated by only one inhibitor, the maximum rate (*G*) is achieved in the absence of the inhibitor, i.e. *g* = *G*. This criterion can be generalized to genes regulated by multiple regulators. Therefore, in practice, we directly randomize the maximum production rate (*G*) instead of the basal production rate (g), and then calculate the value of g according to the above criterion. The ranges of these parameters are summarized in details in S1 Table. The RACIPE randomization procedure allows a gene to have a relative expression ratio of up to 1,000 for two sets of parameters, even when it is not regulated by other genes.

Second, we also assume that, for all the members of the RACIPE model ensemble, each regulatory link in the circuit should have roughly equal chance of being functional or not functional, referred to as the *half-functional* rule. For example, in the case that gene A regulates gene B, all the threshold parameters are selected in such a way that, for the RACIPE ensemble, the level of A at the steady states has roughly 50% chance to be above and 50% chance to be below its threshold level. Otherwise, if the threshold level is too large or too small, the regulatory link is either not functional most of the time or constitutively active, thereby changing the effective circuit topology, and limiting the comprehensive understanding of circuit function (S2 Fig).

To achieve this, we estimate the range of the threshold levels by a mean-field approximation, and use this range to randomly sample the threshold parameters. For a regulatory link from gene A (regulator) to gene B (target), the threshold level *AB*_0_ can be estimated as follows. We first estimate the range of expression of gene A without considering any of its regulators. The A level without regulation satisfies

$$\dot{A}=G-kA,$$ (2)

By randomizing both *G* and *k* by the aforementioned protocol (S1 Table), we generate an ensemble of random models, from which we obtain the distribution of the steady state levels of gene *A* (Fig 2A). To meet the half-functional rule, the median of the threshold level should be chosen to be the median of this distribution. When gene A is regulated by some other genes (i.e. its upstream regulators), we estimate its median threshold level by taking A’s regulators into account, and assume that the levels of all these regulators (*e*.*g*. gene B, C *etc*.) follow the same distribution as an isolated gene (top right panels in Fig 2A section 2). We randomly sample the threshold of every inward regulation from the range of 0.02M to 1.98M, where M is the median of the distribution of an isolated gene. With all of the information, we can again generate a new ensemble of models, from which we calculate the distribution of gene A (bottom panel in Fig 2A section 2) and its median. For every target gene regulated by the gene A, we randomly select the threshold levels of the regulations in the range from 0.02M to 1.98M, where M is the above obtained median level of gene A. The same approach is used to estimate the threshold levels of the other genes. It is worth-noting that this simple estimation strategy works quite well for the cases we have tested (Fig 2B) according to the *half-functional* rule.

In the following, we will first demonstrate the application of RACIPE to some simple toggle-switch-like motifs, then to a set of motifs of coupled toggle-switch circuits, and eventually to a more complex gene regulatory network of EMT. For each case, we will illustrate how we can utilize an ensemble of RACIPE models to identify the dynamic behavior of a gene circuit.

## Results

### RACIPE as an unbiased method to predict robust gene states for a gene circuit

**Fig 3:**
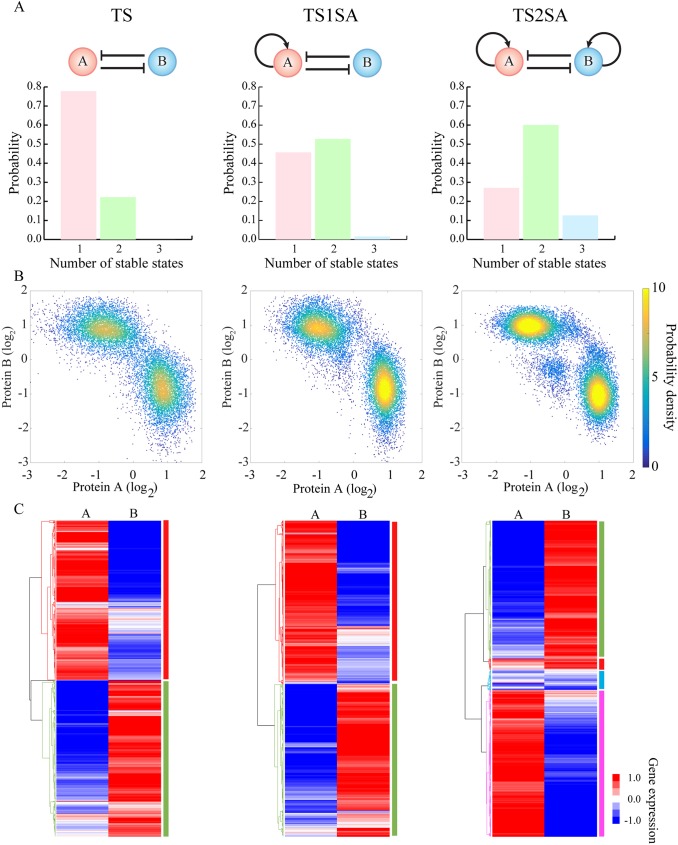
RACIPE identifies robust features of toggle-switch-like motifs. RACIPE was tested on three circuits–a simple toggle-switch (TS, top left) which consists of genes A and B that mutually inhibit each other (solid lines and bars), a toggle-switch with one-sided self-activation (TS1SA) which has an additional self-activation link on gene A, and a toggle-switch with two-sided self-activation (TS2SA) which has additional self-activation links on both genes. **(A)** Probability distributions of the number of stable steady states for each circuit. **(B)** Probability density maps of the gene expression data from all the RACIPE models. Each point represents a stable steady state from a model. For any RACIPE model with multiple stable steady states, all of them are shown in the plot. **(C)** Average linkage hierarchical clustering analysis of the gene expression data from all the RACIPE models using the Euclidean distance. Each column corresponds to a gene, while each row corresponds to a stable steady state from a model. The analysis shows that the gene expression data could be clustered into distinct groups, each of which is associated with a gene state, as highlighted by different colors on the right of the heatmaps.

We first tested RACIPE on several basic toggle-switch-like circuit motifs (Fig 3A). These circuit motifs are considered to be some of the main building blocks of gene regulatory networks[44]. A genetic toggle switch (TS), composed of two mutually inhibitory genes, is commonly considered to function as a bi-stable switch—it allows two stable gene states, each of which is characterized by the dominant expression of one gene. TS has been shown to be a central piece of decision-making modules for cell differentiation in several incidences[45–47].

Here we apply RACIPE to the TS motif. We created an ensemble of 10,000 models (Fig 3A) and we observed that about 20% of models allow two coexisting stable steady states (bi-stability), while the others allow only one steady state (mono-stability). The observation that only a small fraction of TS models works as a bi-stable system is consistent with a previous study[39]. Next, we tested RACIPE on a toggle switch with an extra excitatory auto-regulatory link acting on only one of the genes (a toggle switch with one-sided self-activation, or TS1SA). The circuit motif now has ~ 50% chance of being bi-stable, much larger than the original TS motif. Interestingly, TS1SA also has ~1% chance of having three co-existing stable steady states (tri-stability), so it can potentially act as a three-way switch[44]. Hence, the RACIPE analysis suggests that TS1SA is more robust than TS for functioning as a switch. Moreover, adding excitatory auto-regulatory links on both sides of the TS motif (TS2SA) further increases the likelihood of bi-stability to ~60%, and meanwhile dramatically increases the likelihood of tri-stability to ~13%. This suggests that TS2SA has more of an ability than these other motifs to function as a three-way switch. Indeed, TS2SA has been proposed to be the core decision-making motif for several cell differentiation processes, and many of these processes exhibit multi-stability[45,46]. Thus, the statistical analysis of the ensemble of random models generated by RACIPE can identify the most robust features of a circuit motif.

Another way to utilize RACIPE is to evaluate the possible gene expression patterns of a circuit motif. We can construct a large set of *in silico* gene expression data, consisting of the gene expression levels of the circuit at every stable steady state for each RACIPE model. In the dataset, the columns correspond to the genes and the rows correspond to the stable steady states. For a RACIPE model with multiple stable steady states, we enter the data in multiple rows. The expression dataset takes a form similar to typical experimental microarray data, and so it can be analyzed using common bioinformatics tools. For each of the above two-gene cases, we visualized the expression data by a scatter plot of the levels of the two genes (Fig 3B). Surprisingly, despite large variations in the circuit parameters across the RACIPE model ensemble, the expression data points converge quite well into several robust clusters. For example, the TS circuit data has two distinct clusters, where one has a high expression of gene A while a low expression of gene B and vice versa for the other cluster. The TS2SA circuit has not only the above two clusters but also an additional cluster with intermediate expression of both genes. These patterns have also been observed in previous experimental[29] and theoretical[44,45,48] studies of the same circuits. Interestingly, if we only include models with a fixed number of stable states (e.g. restrict the ensemble to mono-stable models, or bi-stable models), a similar pattern of clusters can still be observed (S3 Fig). These clusters represent distinct patterns of gene expression that the circuit can support, so we will refer to these clusters as “gene states”. These gene states are robust against large perturbations of circuit parameters because the circuit topology restricts possible gene expression patterns. RACIPE in a sense takes advantage of this feature to interrogate the circuit so that we can unbiasedly identify the robust gene states. Since these states may be associated with different cell phenotypes during cell differentiation or cellular decision-making processes, RACIPE can be especially helpful in understanding the regulatory roles of the circuit during transitions among different states.

These simple cases demonstrate the effectiveness of RACIPE in revealing generic properties of circuit motifs. Recall that our basic hypothesis is that the dynamic behaviors of a circuit should be mainly determined by circuit topology, rather than a specific set of parameters. The rich amount of gene expression data generated by RACIPE allows the application of statistical learning methods for the discovery of these robust features. For example, as shown in Fig 3C, we applied unsupervised hierarchical clustering analysis (HCA) to the RACIPE gene expression data, and again we identified similar gene state clusters for each circuit.

**Fig 4:**
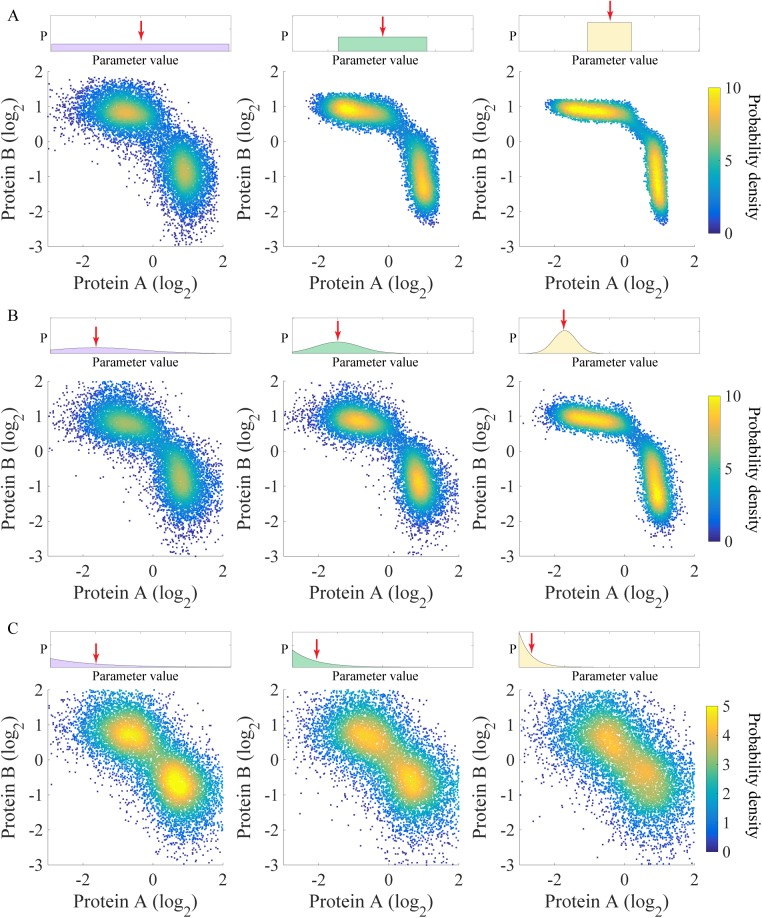
The gene states of the toggle-switch motif are robust against different types of distributions used to sample the parameters. **(A)** Uniform distributions in three different ranges were used to sample the kinetic parameters of the RACIPE models. The top panels show the range of the distribution (left panel: the full range; middle panel: half; right panel: one-fourth). The bottom panels show the probability density maps of the gene expression data from all the RACIPE models. Similarly, panels (B) and (C) show the use of a Gaussian distribution and an exponential distribution, respectively. For the Gaussian distribution (B), its standard deviation was shrunk by a factor of two from left to right. For the Exponential distribution (C), its mean was reduced by a factor of two from left to right. The means of the distributions are indicated by red arrows.

Notably, the predictions of these gene states by RACIPE should be robust against different sampling distributions and different ranges of kinetic parameters. To verify this, we tested on the TS circuit versions of RACIPE created with three different distributions (uniform, Gaussian and exponential distributions) and three different ranges of parameters (Fig 4). Even though the precise shape of gene states appears to be slightly different for the different cases, the number and the locations of these gene states are consistent (Fig 4). For the cases with exponential distribution, in order to reduce the range of the parameters, we decreased the mean of the distribution; therefore, the two gene states become closer (Fig 4). We also found that the changes of the parameter ranges still result in similar gene states (S4 and S5 Figs).

### The application of RACIPE to coupled toggle-switch motifs

**Fig 5:**
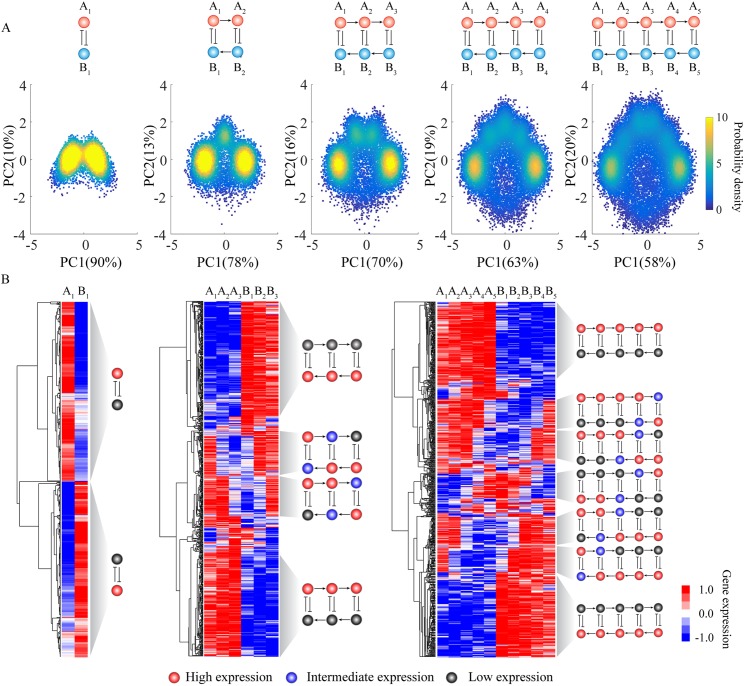
Application of RACIPE to coupled toggle-switch circuits. RACIPE was tested on coupled toggle-switch circuits, as illustrated at the top of the figure. **(A)** 2D probability density map of the RACIPE-predicted gene expression data projected to the 1^st^ and 2^nd^ principal component axes. **(B)** Average linkage hierarchical clustering analysis of the gene expression data from all the RACIPE models using the Euclidean distance. Each column corresponds to a gene, while each row corresponds to a stable steady state. The clustering analysis allows the identification of several robust gene states, whose characteristics were illustrated as circuit cartoons to the right of the heatmaps. The expression levels of each gene in these gene states are illustrated as low (grey), intermediate (blue), or high (red). See S6 and S7 Figs for the definitions.

To evaluate the effectiveness of RACIPE on larger circuits, we further applied the method to circuits with two to five coupled toggle-switch (CTS) motifs (Fig 5). Different from the above simple circuit motifs, the gene expression data obtained by RACIPE for these CTS motifs are now high-dimensional; thus in the scatter plot analysis we projected these data onto the first two principal components by principal component analysis (PCA). For each circuit, we observed distinct gene states from PCA for the RACIPE models (Fig 5A). More interestingly, the number of gene states found via PCA increases by one each time one more toggle switch is added to the circuit. Moreover, we applied HCA to the gene expression data, from which we identified the same gene states as from PCA (Fig 5B). At this stage, we can also assign high (red circles), intermediate (blue circles) or low expression (black circles) to each gene for every gene state. Unlike in Boolean network models, the assignment in RACIPE is based on the distribution of expression pattern from all the models in the ensemble (S6 and S7 Figs).

We can easily understand the meaning of each gene state. In each case, the rightmost cluster in the scatter plot (Fig 5A) corresponds to the topmost cluster in the heatmap (Fig 5B), a state where all the A genes have high expression and all the B genes have low expression. Similarly, the leftmost cluster in the scatter plot corresponds to the bottommost cluster in the heatmap. These two clusters are the most probable ones, and represent the two extreme states of the coupled toggle switch network. As also illustrated in the scatter plots, for circuits with additional toggle switches, these two states separate from each other and the circuit now allows intermediate states. By closely examining these intermediate states, we found that they (from top to bottom) correspond to a cascade of flips of the state of each consecutive toggle switch. This explains why we observe one additional gene state every time we include an additional toggle-switch motif. In addition, intermediate expression levels were frequently observed for genes lying in the middle toggle-switch motifs, instead of those at the edge. The tests on CTS circuits demonstrate again the power of RACIPE in identifying robust features of a complex circuit.

### The application of RACIPE to the EMT circuit

**Fig 6:**
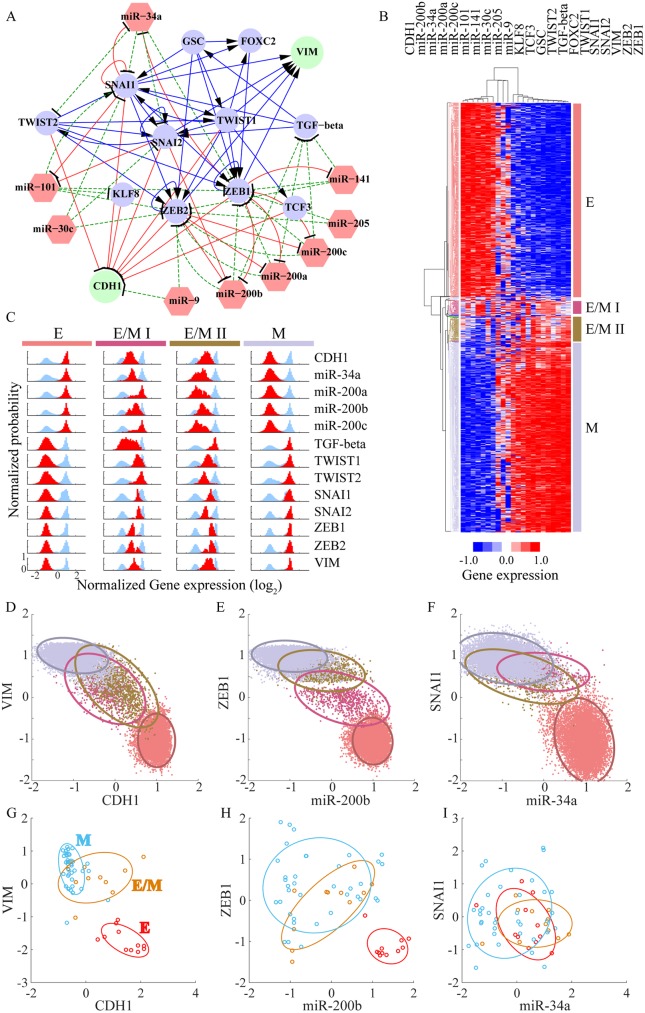
RAICPE identifies multiple EMT cell states from gene network analysis. **(A)** A proposed Epithelial-to-Mesenchymal Transition (EMT) circuit is constructed according to the literature; the circuit consists of 13 transcriptional factors (circles), 9 microRNAs (red hexagons) and 82 regulatory links. The blue solid lines and arrows represent transcriptional activations, the red solid lines and bars represent transcriptional inhibition, and the green dashed lines and bars stand for translational inhibition. Two readout genes CDH1 and VIM are shown as green circles while the other transcriptional factors are shown in blue. **(B)** Average linkage hierarchical clustering analysis of the gene expression data from all the RACIPE models using the Euclidean distance. Each column corresponds to a gene, and each row corresponds to a stable steady state. Four major gene states were identified and highlighted by different colors. According to the expression levels of CDH1 and VIM, the four gene states were associated with epithelial (E in red), mesenchymal (M in grey) and two hybrid epithelial/mesenchymal (E/M I in purple and E/M II in brown) phenotypes. **(C)** The gene expression distribution of each gene state. The gene expression distribution of each gene for all of the RACIPE models is shown in blue, while that for each gene state is shown in red (50 bins are used to calculate the histogram of each distribution). For clarity, each distribution is normalized by its maximum probability. Each row represents a gene and each column represents a gene state. **(D-F)** Gene expression data were projected to either CDH1/VIM, miR-200b/ZEB1, or miR-34a/SNAI1 axes. Different gene states are highlighted by the corresponding colors and enclosed by the ellipses. **(G-I)** Transcriptomics data from the NCI-60 cell lines were projected to either CDH1/VIM, miR-200b/ZEB1, or miR-34a/SNAI1 axes. The NCI-60 cell lines have been grouped into E, E/M and M phenotypes according to the ratio of the protein levels of CDH1 and VIM. Different gene states are highlighted by the corresponding colors and enclosed by the ellipses.

The above examples were used for illustrative purposes and do not immediately reflect any actual biological process. In our last example, we apply RACIPE to a more realistic case, the decision-making circuit of EMT (Fig 6). EMT is crucial for embryonic development, wound healing, and metastasis[49], the last being a major cause for 90% cancer-related deaths[50]. Cells can undergo either a complete EMT to acquire mesenchymal phenotype or partial EMT to attain hybrid E/M phenotype[51,52], which maintains both E and M traits. Transitions among the Epithelial (E), Mesenchymal (M) and hybrid epithelial/mesenchymal (E/M) phenotypes have been widely studied either experimentally or theoretically[52]. Here, we utilized data from the literature and Ingenuity Pathway Analysis (see details in S1 Text) to construct a core gene regulatory circuit model of EMT (Fig 6A), which contains 13 transcriptional factors (TFs), 9 microRNAs (miRs) and 82 regulatory links among them. Among the gene components, two biomarkers–CDH1 and VIM–are commonly used to distinguish different phenotypes during EMT. The circuit is a much-extended version of several previous EMT models[24,25], which consist of only four gene families. It is similar in terms of scale to a recently proposed Boolean model of EMT[53], but as stressed here our models allow for continuous expression levels.

For simplicity, we modeled the EMT circuit with the same approach as above, i.e. all the genetic components were coupled with Hill functions, typical of transcriptional control. This may not be completely accurate for a circuit with different types of regulations, such as the translational regulation by microRNA (miR), but we leave this complication for future study. Notably, although the genome of cancer cells during EMT does not change, the core EMT circuit is still regulated by peripheral genes, epigenetic modifications, and cell signaling, etc. All of these factors contribute to the random perturbations to the kinetic parameters of the 22-node EMT gene regulatory circuit. Even with this simplification, RACIPE can provide insightful information of the EMT regulation. Consistent with what we learned from the test cases, unsupervised HCA of the RACIPE gene expression data can reveal distinct gene states (Fig 6B). Here there are four such states. We can map these gene states to different cell phenotypes possible during EMT–an E phenotype with high expression of the miRs, low expression of TFs, and CDH1^HI^VIM^LO^; a M phenotype with low expression of the miRs, high expressions of TFs, and CDH1^LO^VIM^HI^; and two hybrid E/M phenotypes with intermediate expression of both miRs, TFs and CDH1/VIM. The E/M I state lies closer to the E state, and the E/M II state lies closer to the M state. More intriguingly, we found SNAI1 and SNAI2 become highly expressed in the E/M I phenotype while ZEB1 and ZEB2 are not fully expressed until the E/M II or the M phenotype (Fig 6C), which is a possibility supported by recent experimental results[25].

Moreover, RACIPE can help to find genes of similar function and filter out less important genes in the core circuit. As shown in Fig 6B, genes are grouped into two major clusters according to their expression levels throughout all the RACIPE models–miRs/CDH1 and TFs/VIM. The former genes are highly expressed mainly in E phenotypes while the latter are highly expressed in M phenotypes. We also found three microRNAs (miR-30c, miR-205, and miR-9) to be randomly expressed in the RACIPE models, indicating these three microRNAs are less important to these EMT phenotypes. From the topology of the circuit, we see that these three microRNAs lack feedback regulation and act solely as inputs.

A typical approach taken in cell biology is to use two biomarkers to identify cells of different states in a mixed population by fluorescence-activated cell sorting (FACS). To mimic the analysis, we projected the gene expression data of the RACIPE models onto the two axes of important genes, as shown in the scatter plots in Fig 6D–6F. In all of the three cases, the E and the M phenotypes can be distinguished. However, for the hybrid phenotypes, the E/M I and the E/M II states overlap in the CDH1-VIM plot (Fig 6D). These two hybrid phenotypes can be separated more easily in the ZEB1-miR200b plot (Fig 6E). In the SNAI1-miR34a plot (Fig 6F), however, the two E/M states overlap with the M state. The theoretical prediction that the SNAI1-miR34a axis is less efficient in distinguishing the states is supported by transcriptomics data from the NCI-60 cell lines[54] (Fig 6G–6I). We see here that either VIM-CDH1 or the ZEB1-miR200b axes are indeed better than the SNAI1-miR34a axes in separating different EMT phenotypes. Our results are also consistent with our previous theoretical finding that ZEB1 is more crucial than SNAI1 in the decision-making of EMT[25].

## Discussion

Recently, the rapid development of genomic profiling tools has allowed the mapping of gene regulatory networks. Yet, it remains a challenge to understand the operating mechanisms and the design principles of these networks. Conventional computational modeling methods provide insightful information; however, their prediction power is usually limited by the incompleteness of the network structure and the absence of reliable kinetics. To deal with these issues, we have developed a new computational modeling method, called RACIPE, which allows unbiased predictions of the dynamic behaviors of a complex gene regulatory circuit. Compared to traditional methods, RACIPE uniquely generates an ensemble of models with distinct kinetic parameters. These models can faithfully represent the circuit topology and meanwhile capture the heterogeneity in the kinetics of the genetic regulation. By modeling the dynamics of every RACIPE model, we can utilize statistical analysis tools to identify the robust features of network dynamics. We have successfully tested RACIPE on several theoretical circuit motifs and a proposed core Epithelial-to-Mesenchymal Transition (EMT) gene regulatory circuit. In each circuit, RACIPE is capable of predicting the relevant gene states and providing insights into the regulatory mechanism of the decision-making among gene states.

Unlike other methods that utilize randomization strategies to explore the parameter sensitivity for gene circuit[34–37], RACIPE adopts a more carefully designed sampling strategy to randomize circuit parameters over a wide range, but meanwhile to satisfy the half-functional rule to gain a comprehensive understanding of circuit dynamics. Instead of looking for the sensitivity of the circuit function to parameter variations [34,37] and the parameters best fitting the experimental data[35,36], we focused on uncovering conserved features from the ensemble of RACIPE models. This was carried out by standard statistical learning methods such as hierarchical clustering analysis. We showed the power of RACIPE to predict the robust gene states for a circuit with a given topology. Also, conceptually similar to the mixed-effects models used to describe a cell population for a very simple system [36], i.e. a one-gene transcription without a regulator, RACIPE could be potentially applied to a very large gene circuit to describe the gene expression dynamics of a cell population with an ensemble of models—an aspect we will work on in our future study. Moreover, it is easy to implement gene modifications such as knockdown or overexpression treatments with the RACIPE method to learn the significance of each gene or link in the circuit. Therefore, RACIPE provides a new way to model a gene circuit without knowing the detailed circuit parameters.

Another parameter-independent approach people often use for gene circuit modeling is Boolean network model[55], which digitalizes the gene expression into on and off states and uses logic functions to describe the combinatorial effects of regulators to their targets. Compared with the Boolean network model, RACIPE is a continuous method, so it is not restricted to the on and off values. Instead, RACIPE enables us to find the intermediate levels of gene expressions beyond the on and off states, as we showed in Fig 5B and Fig 6C. From the ensemble of RACIPE models, we can predict the expression distribution of each gene, which can be directly compared with experimental expression data. The comparison will allow us to further refine the core circuit. In addition, in RACIPE, we not only obtain *in silico* gene expression data, but we also have the kinetic parameters for each model. From these parameter data, we can directly compare the parameter distributions for different gene states, from which we can learn the driving parameters that are responsible for the transitions among the states.

To conclude, here we have introduced a new theoretical modeling method, RACIPE, to unbiasedly study the behavior of a core gene regulatory circuit under the presence of intrinsic or extrinsic fluctuations. These fluctuations could represent different signaling environments, epigenetic states, and/or genetic backgrounds of the core circuit and can cause cell-cell heterogeneity in a population. By approximating these fluctuations as variations of the model parameters, RACIPE provides a straightforward way to understand the heterogeneity and to explain further how gene circuits can perform robust functions under such conditions. Moreover, RACIPE uniquely generates a large set *in silico* expression data, which can be directly compared with experimental data using common bioinformatics tools. RACIPE enables the connection of traditional circuit-based bottom-up approach with profiling-based top-down approach. We expect RACIPE to be a powerful method to identify the role of network topology in determining network operating principles.

## Supporting information

S1 Table Ranges of the parameters for randomization. (DOCX)

S1 Text Mathematical models and simulation details. (DOCX)

S1 Fig Application of RACIPE to study oscillatory dynamics of a repressilator gene circuit. **(**A) Illustration of a repressilator circuit with three genes, where each gene represses the next gene in the circuit. (B) Tests of the half-functional rule for all RACIPE models (leftmost panel), the models with stable steady states (middle panel), and the models with stable oscillation (right panel). For the models with stable oscillation, we computed the ratio (x/x_0_) of the mean level of each gene X during oscillation and the threshold (x_0_) for the outward regulations from gene X. The yellow region shows the probability of x/x_0_ > 1 for the models, and the green region shows the probability of x/x_0_ < 1. (C) 2D probability density map of the RACIPE-predicted gene expression data of the models with stable steady states projected to the 1^st^ and 2^nd^ principal component axes. (D) Projection of the oscillatory trajectories of the models with stable oscillations to the same 1^st^ and 2^nd^ principal component axes in (C). (E) The overlapping of the PCA results between (C) and (D).
(TIF)

S2 Fig Tests of several random sampling schemes with and without the half-functional rule. **(**A) Test of the half-functional rule of a toggle-switch with one-sided self-activation where different ranges were used to randomize the threshold parameters. The leftmost panel shows the circuit and the sampling ranges of the threshold parameters by RACIPE. The middle and the rightmost panels show two examples where same ranges of the threshold parameters are used for all regulatory links. (B) Probability distributions of the number of stable steady states for each circuit. (C) Probability density maps of the gene expression data from all the RACIPE models, where the fraction of stable gene expressions in each quadrant is shown.
(TIF)

S3 Fig **Probability density maps of the gene expression data from all the RACIPE models with a fixed number of stable states for TS (A, B), TS1SA (C, D) and TS2SA (E, F and G) motifs.** A, C and E are the maps for mono-stable models, B, D, and F are the maps for bi-stable models while G is for the tri-stable models. For ensembles with different number of stable steady states, the gene state clusters remain the similar (e.g., gene expression patterns, the locations of the clusters from PCA), but the percentage of models in each gene state varies.
(TIF)

S4 Fig The effects of the range of parametric perturbations on the robust gene states of a toggle-switch circuit motif. Each panel shows the probability density map of the gene expression data from all the RACIPE models for a version of RACIPE with a different level of parametric perturbations. **(**A) The range of the production rates was randomized from 1–1000. (B) The range of the degradation rates was randomized from 0.1–10. (C) The range of the fold changes was randomized from 1–1000. In each case, the range of variations is 10 times as large as the original method. The randomization procedure for the rest parameters remains the same as the original RACIPE. Uniform distributions are used to randomize the parameters in these cases.
(TIF)

S5 Fig The effects of Hill coefficients on the robustness of the sampling scheme and gene states of a toggle-switch circuit motif. Test of the half-functional rule (top-panels) and 2D probability density map (bottom-panels) of RACIPE-generated gene expression data are shown for cases where Hill coefficients are randomized with different ranges—A: 1–3; B: 4–6; C: 7–9.
(TIF)

S6 Fig Gene expression distributions of each gene state for the CTS motif with three coupled toggle switches. The gene expression distribution of each gene for all of the RACIPE models is shown in blue, while that for each gene state is shown in red (50 bins for the histogram of each distribution). Below, each row shows the distribution of each gene for every gene state, listed in the same order as Fig 5B. For clarity, each distribution is normalized by its maximum probability. Each column represents a gene and each row represents a gene state. For each state, the expression of a gene could be assigned as a high, intermediate or low level according to the relative location of its distribution (red) with respect to the distribution (blue) for all the RACIPE models.
(TIF)

S7 Fig Gene expression distributions of each gene state for the CTS motif with five coupled toggle switches. The gene expression distribution of each gene for all of the RACIPE models is shown in blue, while that for each gene state is shown in red (50 bins for the histogram of each distribution). Below, each row shows the distribution of each gene for every gene state, listed in the same order as Fig 5B. For clarity, each distribution is normalized by its maximum probability. Each column represents a gene and each row represents a gene state. For each state, the expression of a gene could be assigned as a high, intermediate or low level according to the relative location of its distribution (red) with respect to the distribution (blue) for all the RACIPE models.
(TIF)

## References

[1] Kircher M, Kelso J. High-throughput DNA sequencing—concepts and limitations. BioEssays News Rev Mol Cell Dev Biol. 2010;32: 524–536. 10.1002/bies.200900181 20486139

[2] Lashkari DA, DeRisi JL, McCusker JH, Namath AF, Gentile C, Hwang SY, et al Yeast microarrays for genome wide parallel genetic and gene expression analysis. Proc Natl Acad Sci. 1997;94: 13057–13062. 9371799 10.1073/pnas.94.24.13057 PMC24262

[3] Guo G, Huss M, Tong GQ, Wang C, Li Sun L, Clarke ND, et al Resolution of Cell Fate Decisions Revealed by Single-Cell Gene Expression Analysis from Zygote to Blastocyst. Dev Cell. 2010;18: 675–685. 10.1016/j.devcel.2010.02.012 20412781

[4] Djebali S, Davis CA, Merkel A, Dobin A, Lassmann T, Mortazavi A, et al Landscape of transcription in human cells. Nature. 2012;489: 101–108. 10.1038/nature11233 22955620 PMC3684276

[5] Moignard V, Macaulay IC, Swiers G, Buettner F, Schütte J, Calero-Nieto FJ, et al Characterization of transcriptional networks in blood stem and progenitor cells using high-throughput single-cell gene expression analysis. Nat Cell Biol. 2013;15: 363–372. 10.1038/ncb2709 23524953 PMC3796878

[6] Boyer LA, Lee TI, Cole MF, Johnstone SE, Levine SS, Zucker JP, et al Core transcriptional regulatory circuitry in human embryonic stem cells. Cell. 2005;122: 947–956. 10.1016/j.cell.2005.08.020 16153702 PMC3006442

[7] Yan J, Wang H, Liu Y, Shao C. Analysis of Gene Regulatory Networks in the Mammalian Circadian Rhythm. PLOS Comput Biol. 2008;4: e1000193 10.1371/journal.pcbi.1000193 18846204 PMC2543109

[8] Zhang R, Lahens NF, Ballance HI, Hughes ME, Hogenesch JB. A circadian gene expression atlas in mammals: Implications for biology and medicine. Proc Natl Acad Sci. 2014;111: 16219–16224. 10.1073/pnas.1408886111 25349387 PMC4234565

[9] Smolen P, Baxter DA, Byrne JH. Mathematical Modeling of Gene Networks. Neuron. 2000;26: 567–580. 10896154 10.1016/s0896-6273(00)81194-0

[10] Wang Z, Potoyan DA, Wolynes PG. Molecular stripping, targets and decoys as modulators of oscillations in the NF-κB/IκBα/DNA genetic network. J R Soc Interface. 2016;13: 20160606 10.1098/rsif.2016.0606 27683001 PMC5046959

[11] Narula J, Kuchina A, Lee DD, Fujita M, Süel GM, Igoshin OA. Chromosomal Arrangement of Phosphorelay Genes Couples Sporulation and DNA Replication. Cell. 2015;162: 328–337. 10.1016/j.cell.2015.06.012 26165942 PMC4506695

[12] Huang B, Lu M, Jolly MK, Tsarfaty I, Onuchic J, Ben-Jacob E. The three-way switch operation of Rac1/RhoA GTPase-based circuit controlling amoeboid-hybrid-mesenchymal transition. Sci Rep. 2014;4. 10.1038/srep06449 PMC4171704 25245029

[13] Lau K-Y, Ganguli S, Tang C. Function constrains network architecture and dynamics: A case study on the yeast cell cycle Boolean network. Phys Rev E. 2007;75: 051907. 10.1103/PhysRevE.75.051907 17677098

[14] Zhao L, Sun T, Pei J, Ouyang Q. Mutation-induced protein interaction kinetics changes affect apoptotic network dynamic properties and facilitate oncogenesis. Proc Natl Acad Sci. 2015;112: E4046–E4054. 10.1073/pnas.1502126112 26170328 PMC4522770

[15] Li S, Zhu X, Liu B, Wang G, Ao P. Endogenous molecular network reveals two mechanisms of heterogeneity within gastric cancer. Oncotarget. 2015;6: 13607–13627. 10.18632/oncotarget.3633 25962957 PMC4537037

[16] Lei X, Tian W, Zhu H, Chen T, Ao P. Biological Sources of Intrinsic and Extrinsic Noise in cI Expression of Lysogenic Phage Lambda. Sci Rep. 2015;5. 10.1038/srep13597 PMC4557085 26329725

[17] Ge H, Qian H, Xie XS. Stochastic Phenotype Transition of a Single Cell in an Intermediate Region of Gene State Switching. Phys Rev Lett. 2015;114: 078101 10.1103/PhysRevLett.114.078101 25763973

[18] Kim JK, Josić K, Bennett MR. The relationship between stochastic and deterministic quasi-steady state approximations. BMC Syst Biol. 2015;9: 87 10.1186/s12918-015-0218-3 26597159 PMC4657384

[19] Zhou JX, Samal A, d’Hérouël AF, Price ND, Huang S. Relative stability of network states in Boolean network models of gene regulation in development. Biosystems. 2016;142–143: 15–24. 10.1016/j.biosystems.2016.03.002 26965665 PMC5149109

[20] Liu J, Prindle A, Humphries J, Gabalda-Sagarra M, Asally M, Lee DD, et al Metabolic co-dependence gives rise to collective oscillations within biofilms. Nature. 2015;523: 550–554. 10.1038/nature14660 26200335 PMC4862617

[21] Fei J, Singh D, Zhang Q, Park S, Balasubramanian D, Golding I, et al Determination of in vivo target search kinetics of regulatory noncoding RNA. Science. 2015;347: 1371–1374. 10.1126/science.1258849 25792329 PMC4410144

[22] Meyer P, Cokelaer T, Chandran D, Kim KH, Loh P-R, Tucker G, et al Network topology and parameter estimation: from experimental design methods to gene regulatory network kinetics using a community based approach. BMC Syst Biol. 2014;8: 13 10.1186/1752-0509-8-13 24507381 PMC3927870

[23] Milo R, Jorgensen P, Moran U, Weber G, Springer M. BioNumbers—the database of key numbers in molecular and cell biology. Nucleic Acids Res. 2010;38: D750–D753. 10.1093/nar/gkp889 19854939 PMC2808940

[24] Lu M, Jolly MK, Levine H, Onuchic JN, Ben-Jacob E. MicroRNA-based regulation of epithelial-hybrid-mesenchymal fate determination. Proc Natl Acad Sci. 2013;110: 18144–18149. 10.1073/pnas.1318192110 24154725 PMC3831488

[25] Zhang J, Tian X-J, Zhang H, Teng Y, Li R, Bai F, et al TGF-β-induced epithelial-to-mesenchymal transition proceeds through stepwise activation of multiple feedback loops. Sci Signal. 2014;7: ra91 10.1126/scisignal.2005304 25270257

[26] Steinway SN, Zañudo JGT, Ding W, Rountree CB, Feith DJ, Loughran TP, et al Network modeling of TGFβ signaling in hepatocellular carcinoma epithelial-to-mesenchymal transition reveals joint Sonic hedgehog and Wnt pathway activation. Cancer Res. 2014;74: 5963–5977. 10.1158/0008-5472.CAN-14-0225 25189528 PMC4851164

[27] Tyson JJ. Modeling the cell division cycle: cdc2 and cyclin interactions. Proc Natl Acad Sci. 1991;88: 7328–7332. 1831270 10.1073/pnas.88.16.7328 PMC52288

[28] Li C, Wang J. Landscape and flux reveal a new global view and physical quantification of mammalian cell cycle. Proc Natl Acad Sci. 2014;111: 14130–14135. 10.1073/pnas.1408628111 25228772 PMC4191801

[29] Gardner TS, Cantor CR, Collins JJ. Construction of a genetic toggle switch in Escherichia coli. Nature. 2000;403: 339–342. 10.1038/35002131 10659857

[30] Elowitz MB, Leibler S. A synthetic oscillatory network of transcriptional regulators. Nature. 2000;403: 335–338. 10.1038/35002125 10659856

[31] Milo R, Shen-Orr S, Itzkovitz S, Kashtan N, Chklovskii D, Alon U. Network Motifs: Simple Building Blocks of Complex Networks. Science. 2002;298: 824–827. 10.1126/science.298.5594.824 12399590

[32] Hartwell LH, Hopfield JJ, Leibler S, Murray AW. From molecular to modular cell biology. Nature. 1999;402: C47–C52. 10.1038/35011540 10591225

[33] Alon U. Network motifs: theory and experimental approaches. Nat Rev Genet. 2007;8: 450–461. 10.1038/nrg2102 17510665

[34] Feng X, Hooshangi S, Chen D, Li G, Weiss R, Rabitz H. Optimizing Genetic Circuits by Global Sensitivity Analysis. Biophys J. 2004;87: 2195–2202. 10.1529/biophysj.104.044131 15454422 PMC1304645

[35] Gutenkunst RN, Waterfall JJ, Casey FP, Brown KS, Myers CR, Sethna JP. Universally Sloppy Parameter Sensitivities in Systems Biology Models. PLOS Comput Biol. 2007;3: e189. 10.1371/journal.pcbi.0030189 PMC2000971 17922568

[36] Llamosi A, Gonzalez-Vargas AM, Versari C, Cinquemani E, Ferrari-Trecate G, Hersen P, et al What Population Reveals about Individual Cell Identity: Single-Cell Parameter Estimation of Models of Gene Expression in Yeast. PLOS Comput Biol. 2016;12: e1004706 10.1371/journal.pcbi.1004706 26859137 PMC4747589

[37] Meir E, von Dassow G, Munro E, Odell GM. Robustness, Flexibility, and the Role of Lateral Inhibition in the Neurogenic Network. Curr Biol. 2002;12: 778–786. 12015114 10.1016/s0960-9822(02)00839-4

[38] Prescott AM, McCollough FW, Eldreth BL, Binder BM, Abel SM. Analysis of Network Topologies Underlying Ethylene Growth Response Kinetics. Plant Biophys Model. 2016; 1308. 10.3389/fpls.2016.01308 PMC5003821 27625669

[39] Li Z, Bianco S, Zhang Z, Tang C. Generic properties of random gene regulatory networks. Quant Biol. 2014;1: 253–260. 10.1007/s40484-014-0026-6 PMC4198180 25328770

[40] Ma W, Trusina A, El-Samad H, Lim WA, Tang C. Defining Network Topologies that Can Achieve Biochemical Adaptation. Cell. 2009;138: 760–773. 10.1016/j.cell.2009.06.013 19703401 PMC3068210

[41] Kanehisa M, Goto S. KEGG: kyoto encyclopedia of genes and genomes. Nucleic Acids Res. 2000;28: 27–30. 10592173 10.1093/nar/28.1.27 PMC102409

[42] Ashburner M, Ball CA, Blake JA, Botstein D, Butler H, Cherry JM, et al Gene ontology: tool for the unification of biology. The Gene Ontology Consortium. Nat Genet. 2000;25: 25–29. 10.1038/75556 10802651 PMC3037419

[43] Lee W-P, Tzou W-S. Computational methods for discovering gene networks from expression data. Brief Bioinform. 2009;10: 408–423. 10.1093/bib/bbp028 19505889

[44] Lu M, Jolly MK, Gomoto R, Huang B, Onuchic J, Ben-Jacob E. Tristability in Cancer-Associated MicroRNA-TF Chimera Toggle Switch. J Phys Chem B. 2013;117: 13164–13174. 10.1021/jp403156m 23679052

[45] Wang J, Zhang K, Xu L, Wang E. Quantifying the Waddington landscape and biological paths for development and differentiation. Proc Natl Acad Sci. 2011;108: 8257–8262. 10.1073/pnas.1017017108 21536909 PMC3100956

[46] Huang S. Hybrid T-Helper Cells: Stabilizing the Moderate Center in a Polarized System. PLOS Biol. 2013;11: e1001632 10.1371/journal.pbio.1001632 23976879 PMC3747982

[47] Sha W, Moore J, Chen K, Lassaletta AD, Yi C-S, Tyson JJ, et al Hysteresis drives cell-cycle transitions in Xenopus laevis egg extracts. Proc Natl Acad Sci. 2003;100: 975–980. 10.1073/pnas.0235349100 12509509 PMC298711

[48] Huang S, Guo Y-P, May G, Enver T. Bifurcation dynamics in lineage-commitment in bipotent progenitor cells. Dev Biol. 2007;305: 695–713. 10.1016/j.ydbio.2007.02.036 17412320

[49] Thiery JP, Acloque H, Huang RYJ, Nieto MA. Epithelial-Mesenchymal Transitions in Development and Disease. Cell. 2009;139: 871–890. 10.1016/j.cell.2009.11.007 19945376

[50] Gupta GP, Massagué J. Cancer metastasis: building a framework. Cell. 2006;127: 679–695. 10.1016/j.cell.2006.11.001 17110329

[51] Nieto MA, Huang RY-J, Jackson RA, Thiery JP. EMT: 2016. Cell. 2016;166: 21–45. 10.1016/j.cell.2016.06.028 27368099

[52] Jolly MK, Boareto M, Huang B, Jia D, Lu M, Ben-Jacob E, et al Implications of the Hybrid Epithelial/Mesenchymal Phenotype in Metastasis. Front Oncol. 2015;5. 10.3389/fonc.2015.00155 PMC4507461 26258068

[53] Steinway SN, Zañudo JGT, Ding W, Rountree CB, Feith DJ, Loughran TP, et al Network modeling of TGFβ signaling in hepatocellular carcinoma epithelial-to-mesenchymal transition reveals joint Sonic hedgehog and Wnt pathway activation. Cancer Res. 2014;74: 5963–5977. 10.1158/0008-5472.CAN-14-0225 25189528 PMC4851164

[54] Reinhold WC, Sunshine M, Liu H, Varma S, Kohn KW, Morris J, et al CellMiner: A Web-Based Suite of Genomic and Pharmacologic Tools to Explore Transcript and Drug Patterns in the NCI-60 Cell Line Set. Cancer Res. 2012;72: 3499–3511. 10.1158/0008-5472.CAN-12-1370 22802077 PMC3399763

[55] Albert I, Thakar J, Li S, Zhang R, Albert R. Boolean network simulations for life scientists. Source Code Biol Med. 2008;3: 16 10.1186/1751-0473-3-16 19014577 PMC2603008

