# Supplementary material for: Interrogating the topological robustness of gene regulatory circuits by randomization
Source: PLoS Comput Biol. 2017 Mar 31;13(3):e1005456. doi: 10.1371/journal.pcbi.1005456 (PMC5391964; doi:10.1371/journal.pcbi.1005456)
Supplement: S1 Table — (DOCX) [file pcbi.1005456.s001.docx]

**S1 Table.** **Ranges of the parameters for randomization**

| Parameter | Range^#^ |
| --- | --- |
| Maximum production rate () | 1-100 |
| Degradation rate () | 0.1-1 |
| Fold change ()^*^ | 1-100 |
| Threshold ($X_{0}$) | Depend on inward regulations |
| Cooperativity of the regulation () | 1, 2, 3, 4, 5, 6 |

^#^Units: the range of values for each parameter may have different meanings/units for different organisms. For example, the maximum production rate (G) can be 10^2^ – 10^4^ copies per hour per cell for E. *Coli*, and it can be 10^3^ - 10^5^ copies per hour per cell for H. *sapiens*. The degradation rate (k) can be 0.1 – 1 hour^-1^ for E. *Coli*, and it can be 0.01 – 0.1 hour^-1^ for H. *sapiens* (data inferred from bionumbers.org[1]). In the current RACIPE method, the exact unit is not crucial to the results, because the gene expression data for all the RACIPE models were normalized before further analysis.

^*^For inhibition, fold change ranges from 0.01 to 1, but a uniform distribution is sampled for the inverse of .

**References**

1. Milo R, Jorgensen P, Moran U, Weber G, Springer M. BioNumbers—the database of key numbers in molecular and cell biology. Nucleic Acids Res. 2010;38: D750–D753. doi:10.1093/nar/gkp889
