# Supplementary material for: Interrogating the topological robustness of gene regulatory circuits by randomization
Source: PLoS Comput Biol. 2017 Mar 31;13(3):e1005456. doi: 10.1371/journal.pcbi.1005456 (PMC5391964; doi:10.1371/journal.pcbi.1005456)
Supplement: S1 Text — (DOCX) [file pcbi.1005456.s002.docx]

**S1 Text. Mathematical models and simulation details**

**Mathematical models for the gene regulatory circuits** The circuits, including simple toggle-switch-like circuit motifs, coupled toggle-switch (CTS) motifs, and the EMT circuit, were modeled by the deterministic rate equations as follows:

Toggle-switch circuit (TS):

, (1)

Toggle-switch circuit with one-sided self-activation (TS1SA):

, (2)

Toggle-switch circuit with two-sided self-activation (TS2SA):

, (3)

Coupled toggle-switch (CTS) motif with n toggle switches:

, (4)

EMT circuit (see section 3 for the details of the circuit construction):

, (5)

where $G_{i}$ represents the maximum production rate of gene i, and $k_{i}$ represents the degradation rate of gene i. For the regulatory link (either activation or inhibition) from gene i to gene j, $ij_{0}$ represents the threshold level of gene i, $n_{ij}$ is the Hill coefficient, and $\lambda_{ij}$ is the maximum fold change of the gene j level caused by gene i. $\lambda_{ij}$ < 1 for an inhibitory regulation, referred to as $\lambda_{ij}^{-}$, while $\lambda_{ij}$ > 1 for an excitatory regulation, referred to as $\lambda_{ij}^{+}$. For the simple gene circuits (TS, TS1SA, TS2SA), A and B stand for the expression levels of the two transcription factors. The notation for each gene in the CTS motifs is shown in Fig. 5A. For the model of the EMT circuit, X_i_ (i = 1, 2 … 22) stand for the expression levels of FOXC2, ZEB1, KLF8, CDH1, miR-101, ZEB2, SNAI1, miR-141, TGF-beta, miR-200a, miR-200b, miR-200c, miR-205, miR-30c, SNAI2, miR-34a, TWIST2, miR-9, VIM, TWIST1, TCF3, GSC, respectively.

**Normalization of gene expression data** From an ensemble of the RACIPE models, we collected a large set of gene expression data, very similar to those obtained in experiment. Therefore, we can apply tools for data analysis to these computationally generated data. Here, we processed the computational expression data by a standard normalization method before we performed clustering analysis. The gene expression levels from the models are normalized by first log transformation and standardization, i.e.

, (6)

**Cluster analysis** We performed cluster analysis on the normalized gene expression data from the RACIPE models as shown in Fig. 3C, Fig. 5B and Fig. 6B. Each column represents a gene, and each row represents a stable steady state of the circuit for a particular RACIPE model. For RACIPE models with more than one stable state, the gene expression profiles for all of the stable states were listed in multiple rows. We applied average linkage hierarchical clustering analysis using Euclidean distance by Cluster 3.0[2] and the results were visualized by JavaTreeview[3]. The cutoff distance was chosen interactively so that major clusters can be identified and each cluster has a distinct gene expression pattern. These clusters correspond to different gene states for the circuit. Principal component analysis was performed on the same data by using “pca” function in Matlab/2014b (Fig. 5A). The major gene clusters can be readily recognizable from the probability density map projected onto the first two principal component axes.

**Analysis of EMT gene expression data from NCI-60 cell lines** The gene transcript z scores of CDH1, VIM, ZEB1, miR-200b, SNAI, miR-34a were downloaded from Cellminer[4] and categorized into epithelial, hybrid E/M and mesenchymal sets based on the ratio of the E-cadherin and Vimentin levels[5].

**Construction of a large EMT decision making circuit**

In our previous studies[6,7], we constructed a coarse-grained core gene regulatory circuit of EMT, consisting of two transcription factor (TF) families (SNAI and ZEB) and two microRNA (miR) families (miR-34 and miR-200). In addition, we also included an input node, representing the integration of multiple cell signaling pathways, such as HGF, NF-κB, WNT, TGF-β and HIF1-α, into SNAI. The outputs of the circuit are two commonly used biomarkers of EMT – CDH1 and VIM.

Here, we expanded the size of the EMT circuit on the basis of the core EMT module as follows. For each gene family, we considered individual members as separate nodes: SNAI1, SNAI2, TWIST1, TWIST2 for the SNAI family, ZEB1 and ZEB2 for the ZEB family, miR-200a, miR-200b, miR-200c, miR-141 and miR-205 for the miR-200 family, and miR-34a for the miR-34 family. The input and output nodes, TGF-beta, CDH1 and VIM, were explicitly included in the new circuit model. We also included additional genes (FOXC2, KLF8, miR-101, miR-30c, miR-9, TCF3 and GSC) and the directed interactions among all the gene components according to recent experimental evidences[8,9], theoretical studies[10–12] and IPA(IPA®, QIAGEN Redwood City, www.qiagen.com/ingenuity).

**References**

1. Eisen MB, Spellman PT, Brown PO, Botstein D. Cluster analysis and display of genome-wide expression patterns. Proc Natl Acad Sci. 1998;95: 14863–14868.

2. Saldanha AJ. Java Treeview--extensible visualization of microarray data. Bioinforma Oxf Engl. 2004;20: 3246–3248. doi:10.1093/bioinformatics/bth349

3. Reinhold WC, Sunshine M, Liu H, Varma S, Kohn KW, Morris J, et al. CellMiner: A Web-Based Suite of Genomic and Pharmacologic Tools to Explore Transcript and Drug Patterns in the NCI-60 Cell Line Set. Cancer Res. 2012;72: 3499–3511. doi:10.1158/0008-5472.CAN-12-1370

4. Park S-M, Gaur AB, Lengyel E, Peter ME. The miR-200 family determines the epithelial phenotype of cancer cells by targeting the E-cadherin repressors ZEB1 and ZEB2. Genes Dev. 2008;22: 894–907. doi:10.1101/gad.1640608

5. Lu M, Jolly MK, Levine H, Onuchic JN, Ben-Jacob E. MicroRNA-based regulation of epithelial-hybrid-mesenchymal fate determination. Proc Natl Acad Sci. 2013;110: 18144–18149. doi:10.1073/pnas.1318192110

6. Jolly MK, Boareto M, Huang B, Jia D, Lu M, Ben-Jacob E, et al. Implications of the Hybrid Epithelial/Mesenchymal Phenotype in Metastasis. Front Oncol. 2015;5. doi:10.3389/fonc.2015.00155

7. Paranjape AN, Soundararajan R, Werden SJ, Joseph R, Taube JH, Liu H, et al. Inhibition of FOXC2 restores epithelial phenotype and drug sensitivity in prostate cancer cells with stem-cell properties. Oncogene. 2016; doi:10.1038/onc.2015.498

8. Lamouille S, Xu J, Derynck R. Molecular mechanisms of epithelial-mesenchymal transition. Nat Rev Mol Cell Biol. 2014;15: 178–196. doi:10.1038/nrm3758

9. Steinway SN, Zañudo JGT, Ding W, Rountree CB, Feith DJ, Loughran TP, et al. Network modeling of TGFβ signaling in hepatocellular carcinoma epithelial-to-mesenchymal transition reveals joint Sonic hedgehog and Wnt pathway activation. Cancer Res. 2014;74: 5963–5977. doi:10.1158/0008-5472.CAN-14-0225

10. Steinway SN, Zañudo JGT, Michel PJ, Feith DJ, Loughran TP, Albert R. Combinatorial interventions inhibit TGFβ-driven epithelial-to-mesenchymal transition and support hybrid cellular phenotypes. Npj Syst Biol Appl. 2015;1: 15014. doi:10.1038/npjsba.2015.14

11. Cohen DPA, Martignetti L, Robine S, Barillot E, Zinovyev A, Calzone L. Mathematical Modelling of Molecular Pathways Enabling Tumour Cell Invasion and Migration. PLOS Comput Biol. 2015;11: e1004571. doi:10.1371/journal.pcbi.1004571
