# Supplementary figures and images for: Interrogating the topological robustness of gene regulatory circuits by randomization
Source: PLoS Comput Biol. 2017 Mar 31;13(3):e1005456. doi: 10.1371/journal.pcbi.1005456 (PMC5391964; doi:10.1371/journal.pcbi.1005456)

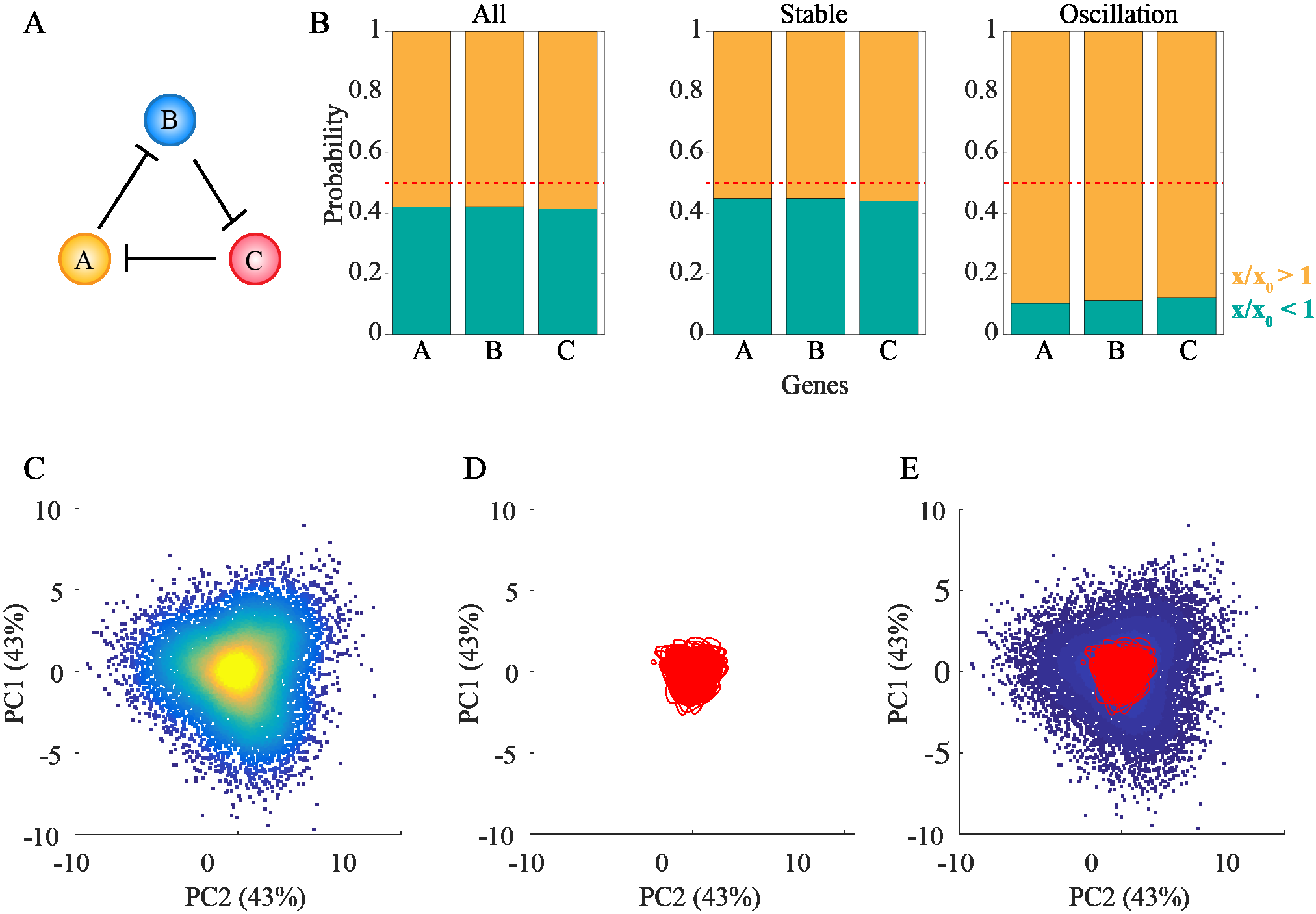

Supplement: S1 Fig — (A) Illustration of a repressilator circuit with three genes, where each gene represses the next gene in the circuit. (B) Tests of the half-functional rule for all RACIPE models (leftmost panel), the models with stable steady states (middle panel), and the models with stable oscillation (right panel). For the models with stable oscillation, we computed the ratio (x/x0) of the mean level of each gene X during oscillation and the threshold (x0) for the outward regulations from gene X. The yellow region shows the probability of x/x0 > 1 for the models, and the green region shows the probability of x/x0 < 1. (C) 2D probability density map of the RACIPE-predicted gene expression data of the models with stable steady states projected to the 1st and 2nd principal component axes. (D) Projection of the oscillatory trajectories of the models with stable oscillations to the same 1st and 2nd principal component axes in (C). (E) The overlapping of the PCA results between (C) and (D). (TIF) [file pcbi.1005456.s003.tif]

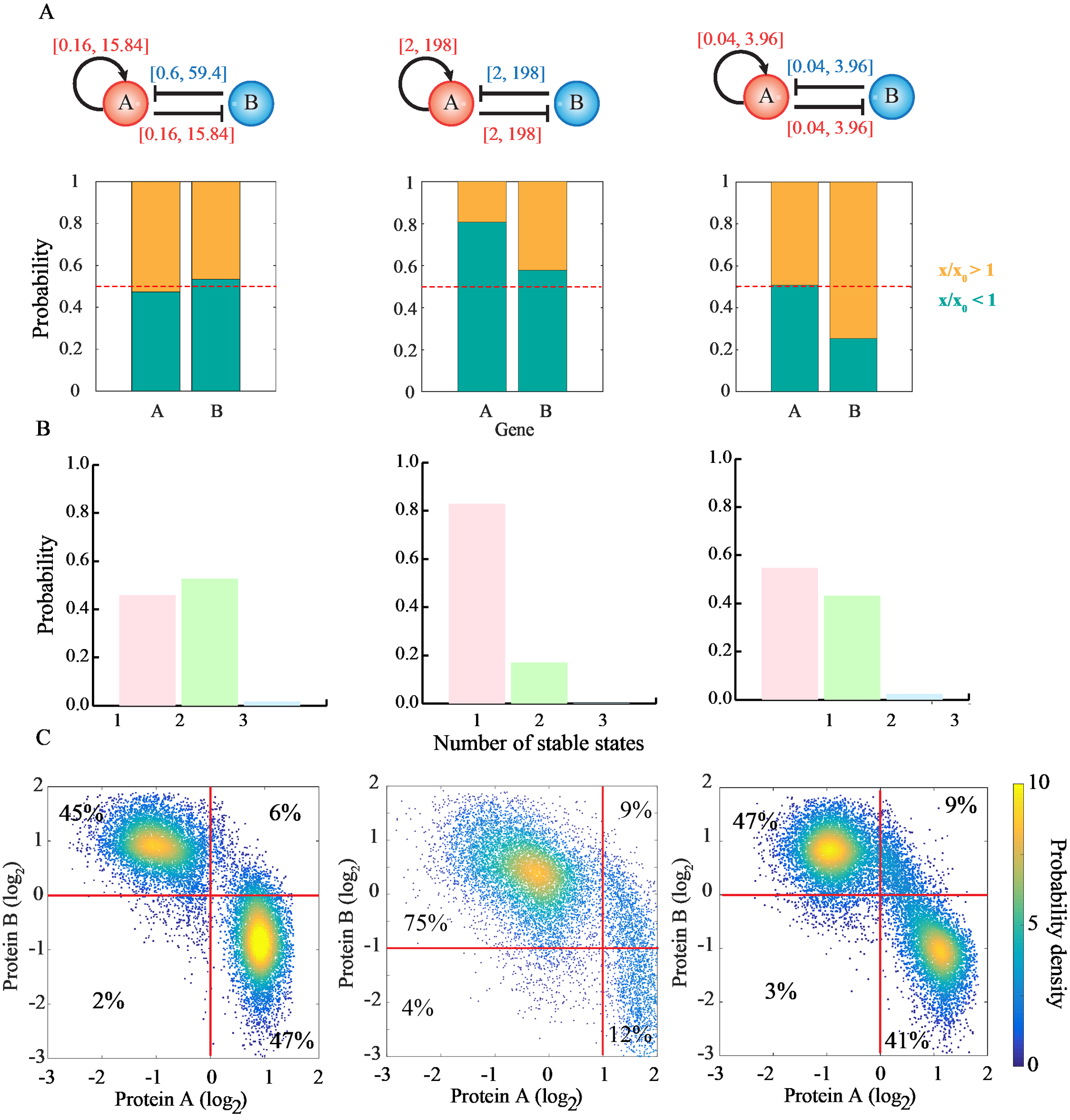

Supplement: S2 Fig — (A) Test of the half-functional rule of a toggle-switch with one-sided self-activation where different ranges were used to randomize the threshold parameters. The leftmost panel shows the circuit and the sampling ranges of the threshold parameters by RACIPE. The middle and the rightmost panels show two examples where same ranges of the threshold parameters are used for all regulatory links. (B) Probability distributions of the number of stable steady states for each circuit. (C) Probability density maps of the gene expression data from all the RACIPE models, where the fraction of stable gene expressions in each quadrant is shown. (TIF) [file pcbi.1005456.s004.tif]

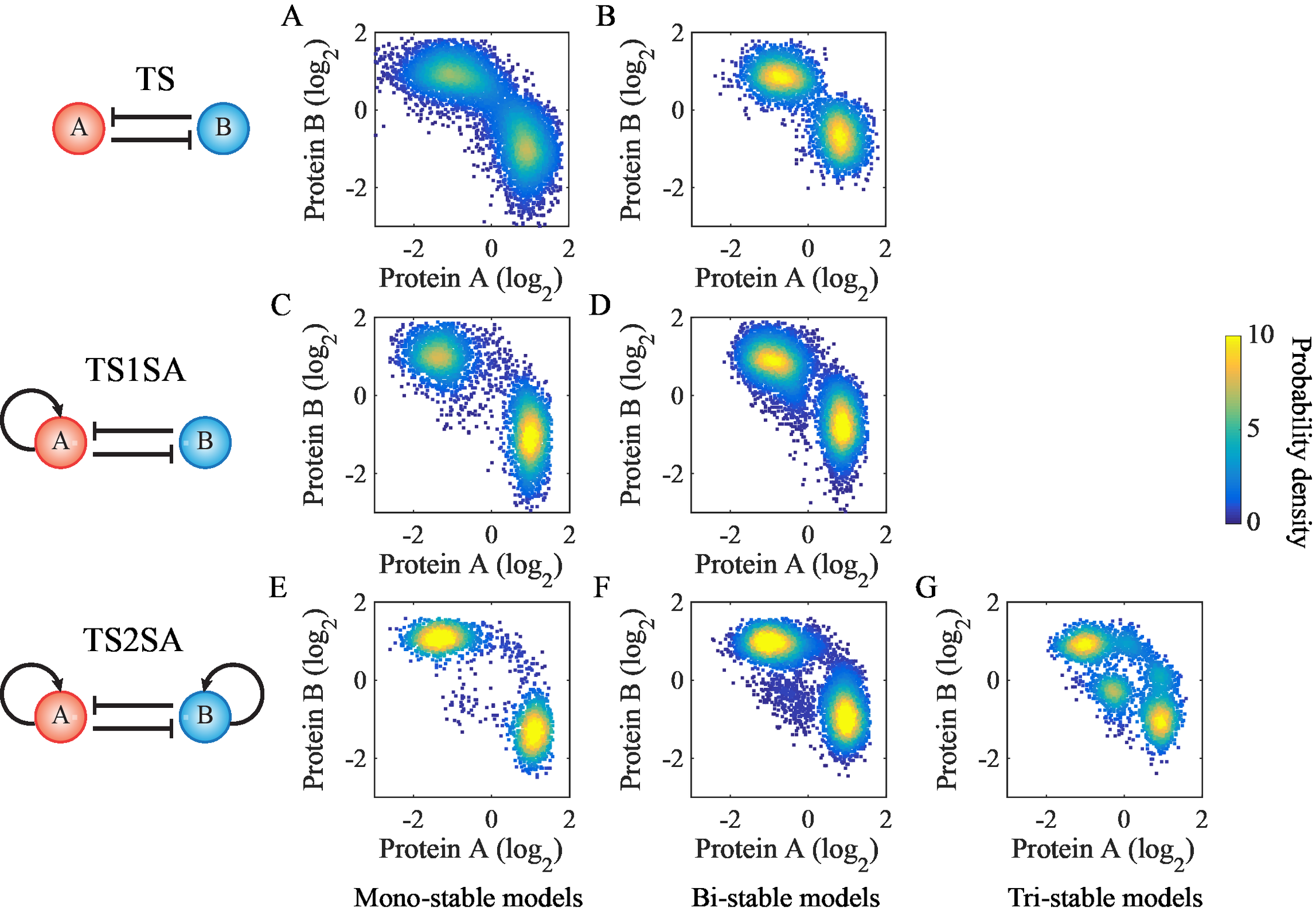

Supplement: S3 Fig — Probability density maps of the gene expression data from all the RACIPE models with a fixed number of stable states for TS (A, B), TS1SA (C, D) and TS2SA (E, F and G) motifs. A, C and E are the maps for mono-stable models, B, D, and F are the maps for bi-stable models while G is for the tri-stable models. For ensembles with different number of stable steady states, the gene state clusters remain the similar (e.g., gene expression patterns, the locations of the clusters from PCA), but the percentage of models in each gene state varies. (TIF) [file pcbi.1005456.s005.tif]

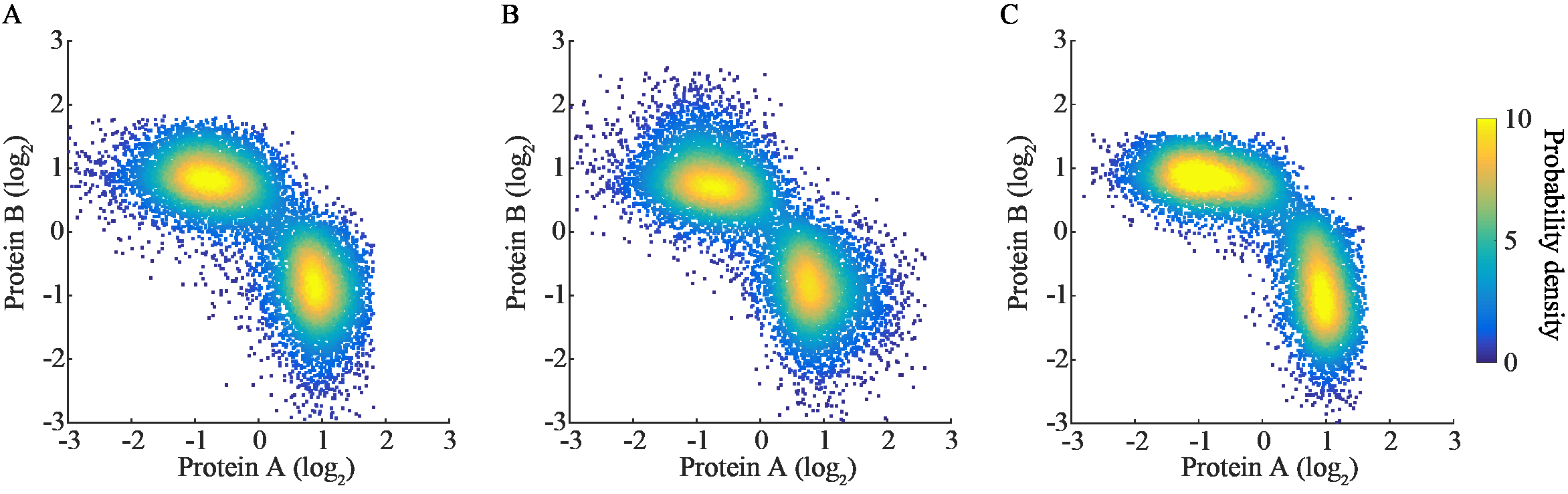

Supplement: S4 Fig — Each panel shows the probability density map of the gene expression data from all the RACIPE models for a version of RACIPE with a different level of parametric perturbations. (A) The range of the production rates was randomized from 1–1000. (B) The range of the degradation rates was randomized from 0.1–10. (C) The range of the fold changes was randomized from 1–1000. In each case, the range of variations is 10 times as large as the original method. The randomization procedure for the rest parameters remains the same as the original RACIPE. Uniform distributions are used to randomize the parameters in these cases. (TIF) [file pcbi.1005456.s006.tif]

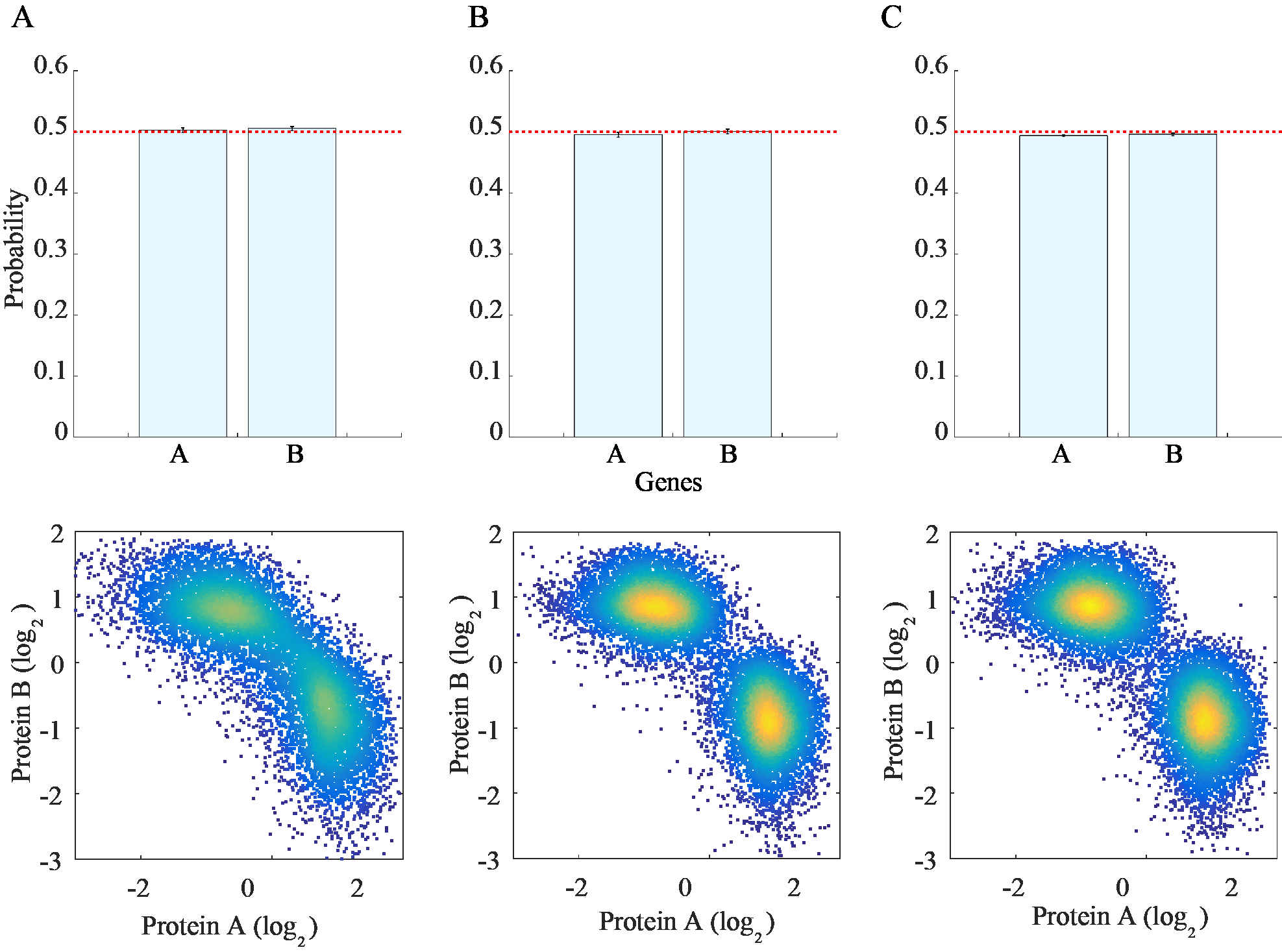

Supplement: S5 Fig — Test of the half-functional rule (top-panels) and 2D probability density map (bottom-panels) of RACIPE-generated gene expression data are shown for cases where Hill coefficients are randomized with different ranges—A: 1–3; B: 4–6; C: 7–9. (TIF) [file pcbi.1005456.s007.tif]

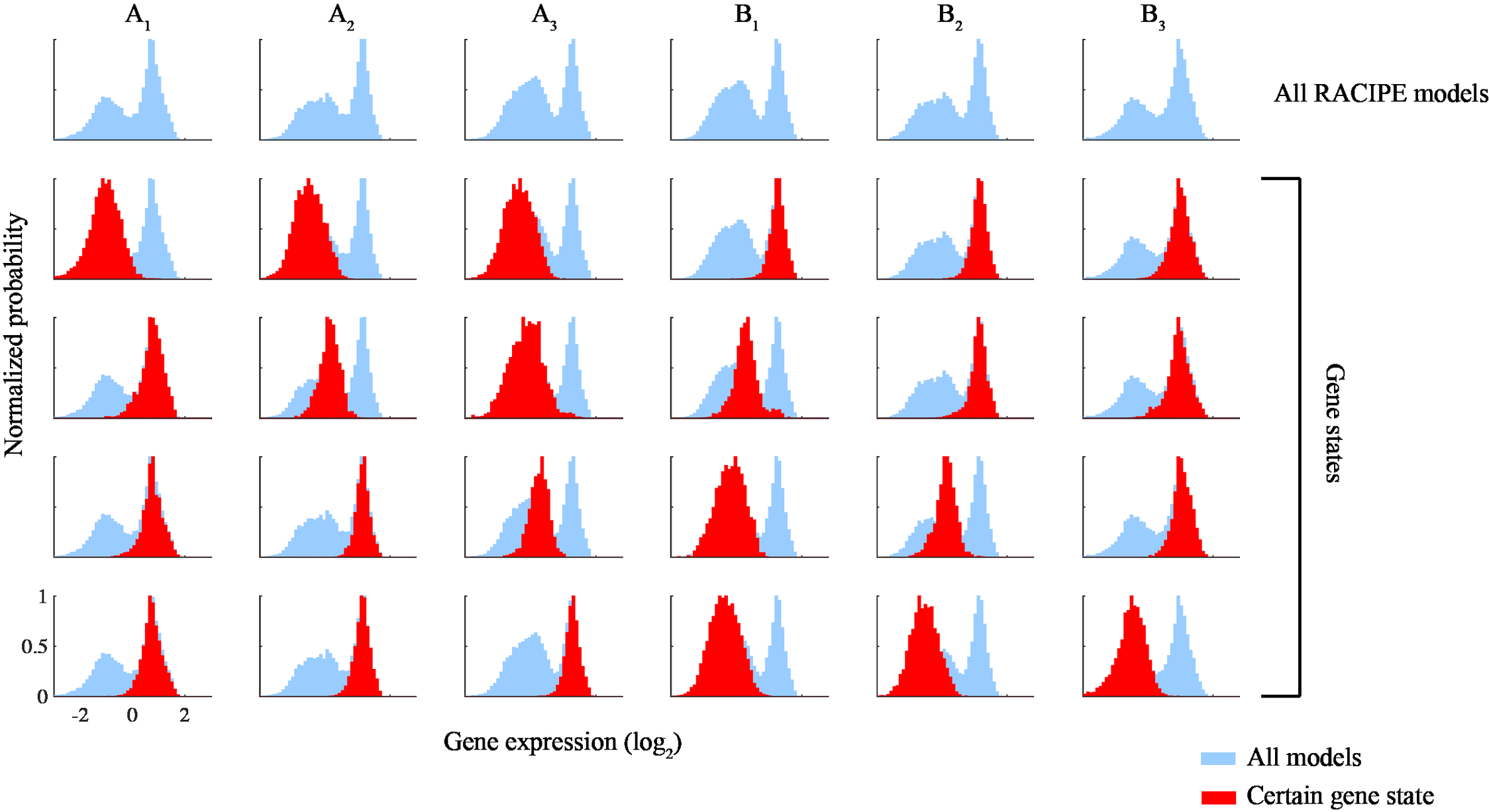

Supplement: S6 Fig — The gene expression distribution of each gene for all of the RACIPE models is shown in blue, while that for each gene state is shown in red (50 bins for the histogram of each distribution). Below, each row shows the distribution of each gene for every gene state, listed in the same order as Fig 5B. For clarity, each distribution is normalized by its maximum probability. Each column represents a gene and each row represents a gene state. For each state, the expression of a gene could be assigned as a high, intermediate or low level according to the relative location of its distribution (red) with respect to the distribution (blue) for all the RACIPE models. (TIF) [file pcbi.1005456.s008.tif]

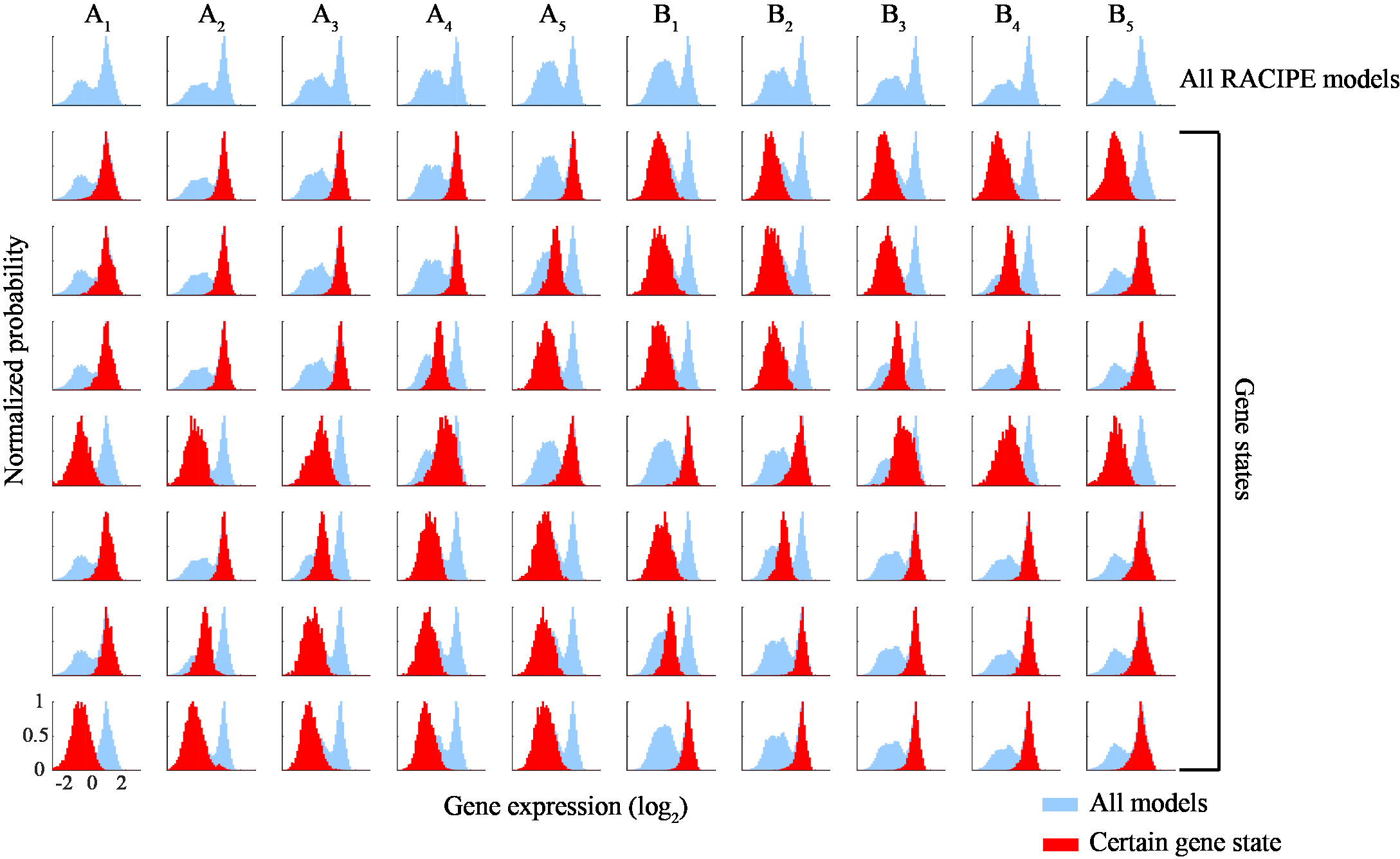

Supplement: S7 Fig — The gene expression distribution of each gene for all of the RACIPE models is shown in blue, while that for each gene state is shown in red (50 bins for the histogram of each distribution). Below, each row shows the distribution of each gene for every gene state, listed in the same order as Fig 5B. For clarity, each distribution is normalized by its maximum probability. Each column represents a gene and each row represents a gene state. For each state, the expression of a gene could be assigned as a high, intermediate or low level according to the relative location of its distribution (red) with respect to the distribution (blue) for all the RACIPE models. (TIF) [file pcbi.1005456.s009.tif]
